# Modelling Japanese encephalitis virus transmission dynamics and human exposure in a Cambodian rural multi-host system

**DOI:** 10.1371/journal.pntd.0010572

**Published:** 2022-07-11

**Authors:** Héléna Ladreyt, Véronique Chevalier, Benoit Durand

**Affiliations:** 1 International Center of Research in Agriculture for Development (CIRAD), UMR ASTRE, Montpellier, France; 2 CIRAD, UMR ASTRE, Antananarivo, Madagascar; 3 Epidemiology and Public Health Unit, Institut Pasteur du Cambodge, Phnom Penh, Cambodia; 4 Epidemiology and Clinical Research Unit, Institut Pasteur de Madagascar, Antananarivo, Madagascar; 5 Epidemiology Unit, Laboratory for Animal Health, French Agency for Food, Environmental and Occupational Health and Safety (ANSES), University Paris-Est, Maisons-Alfort, France; Australian Red Cross Lifelood, AUSTRALIA

## Abstract

Japanese encephalitis (JE) is a vector-borne zoonosis and the leading cause of human viral encephalitis in Asia. Its transmission cycle is usually described as involving wild birds as reservoirs and pigs as amplifying hosts. JE is endemic in Cambodia, where it circulates in areas with low pig densities (<70 pigs per km^2^), and could be maintained in a multi-host system composed of pigs, but also poultry as competent hosts, and dogs, cattle and humans as non-competent hosts. We used a mathematical model representing Japanese encephalitis virus (JEV) transmission in a traditional Cambodian village that we calibrated with field data collected in 3 districts of Kandal province, Cambodia. First, R_0_ calculations allowed us to assess the capacity of the epidemiological system to be invaded by JEV and sustain virus transmission in villages in the 3 districts, and we predicted human exposure at the epidemiological equilibrium, based on simulations. Changes in spatial density of livestock, in agricultural practices, and epizootics (e.g., African swine fever), can profoundly alter the composition of host communities, which could affect JEV transmission and its impact on human health. In a second step, we then used the model to analyse how host community composition affected R_0_ and the predicted human exposure. Lastly, we evaluated the potential use of dog JE seroprevalence as an indicator of human exposure to JEV. In the modeled villages, the calculated R_0_ ranged from 1.07 to 1.38. Once the equilibrium reached, predicted annual probability of human exposure ranged from 9% to 47%, and predicted average age at infection was low, between 2 and 11 years old, highlighting the risk of severe forms of JEV infection and the need to intensify child immunization. According to the model, increasing the proportion of competent hosts induced a decrease in age at infection. The simulations also showed that JEV could invade a multi-host system with no pigs, reinforcing the assumption of poultry acting as reservoirs. Finally, the annual human exposure probability appeared linearly correlated with dog seroprevalence, suggesting that in our specific study area, dog seroprevalence would be a good proxy for human exposure.

## Author summary

Japanese encephalitis virus (JEV) is endemic in Cambodia and remains the most common cause of acute viral encephalitis, particularly in children and adolescents. The traditionally described cycle of JEV, involving wild birds as reservoirs, pigs as amplifying hosts and *Culex* mosquitoes as vectors is questioned, with increasing evidence of a more complex multi-host system involved in areas where densities of pigs are low. In Cambodia, the infection could be maintained in a multi-host system consisting of pigs and poultry as competent hosts, and dogs, cattle and humans as non-competent hosts. We defined a compartmental dynamic model of JEV transmission in a multi-host system representing a rural Cambodian village, to predict human exposure to JEV in the studied area, and to analyse how host community composition may affect human exposure and R_0_ value. Our theoretical approach showed that variations of the composition of the multi-host system may have an impact on human exposure to JEV, and thus on the disease burden in humans, especially in young children. Besides children vaccination in JEV endemic areas, a proper evaluation of the impact on human health is needed to target prevention actions and reduce JEV burden in Cambodia.

## Introduction

Japanese encephalitis virus (JEV) is the leading cause of human acute viral encephalitis in Asia. Even if the global burden of JEV on human health is difficult to evaluate, this vaccine-preventable vector-borne zoonosis was estimated to have caused around 100,000 cases and more than 25,000 deaths in 2015 [1], with an observed case fatality rate up to 30%. Severe neurological sequelae may persist in 30% to more than 50% of survivors [2–6]. Approximately three quarters of cases concern children and JE remains a substantial public health issue even in areas where human vaccination programs are implemented [7]. Furthermore, JEV exposure figures are likely to be underestimated due to under-detection and under-diagnosis of acute encephalitis, particularly in developing countries, as well as cross-reactivity of serological tests with other flaviviruses, especially dengue virus [8,9].

It is commonly accepted that JEV is transmitted from Ardeid birds (wild reservoir hosts) or domestic pigs (amplifying hosts) to humans through the bites of *Culex*, and probably some *Aedes* mosquitoes [10,11]. However, JEV circulates in areas with low densities of domestic pigs or Ardeid birds, such as in Singapore, where its circulation was detected in sentinel poultry years after pig farming was phased out [12]. Thus, the epidemiology of JE may differ from one region to another [13,14]. Domestic chickens and ducks have been shown to be exposed to JEV in several regions in Asia [8,15–18]. A serosurvey conducted in Kandal province, Cambodia, showed that JEV force of infection, *i*.*e*. the instantaneous probability to become infected, exerted on ducks was comparable to that exerted on pigs [16]. Chicks and ducklings also develop significant viremia after being bitten by infected mosquitoes [19–22] and may be able to infect susceptible mosquitoes feeding on them [23,24], suggesting that young poultry may be competent hosts, *i*.*e*. able to transmit JEV to vectors [25]. However, no study has yet fully investigated the potential of poultry as competent hosts for JEV under natural conditions.

*Culex spp*. mosquitoes, the main vectors of JEV, are opportunistic and can feed on various host species, depending on local abundance of hosts and vector species-specific intrinsic factors [26–28]. These hosts can be competent, such as pigs, waterfowl and poultry [8,12,29,30], or not, such as cattle, dogs, humans and horses, which may “dilute” mosquito bites [31–34]. The basic reproduction number (R_0_) is the expected number of secondary cases generated by a primary case in an entirely susceptible population [35]. R_0_ is the indicator commonly used to measure whether or not a pathogen can invade a population. R_0_ greater than 1 is a necessary but not sufficient condition for self-sustained transmission, which also requires a renewal of the susceptible and competent host pool. Variations in the composition of the multi-host system may thus affect R_0_ value.

Japanese encephalitis is endemic in Cambodia, and remains the most common cause of acute encephalitis, particularly in children and adolescents [33,36]. Cambodia is a predominantly rural country, although the density of domestic pigs is low (around 20 pigs per km^2^ on average) compared to other countries where JEV is circulating, such as Japan, China or Vietnam where densities can reach 700 pigs per km^2^ [13,37–39]. In Kandal province, a rural area of Cambodia surrounding the capital Phnom Penh, the majority of the livestock is raised in backyards, with close proximity between pigs, chickens, ducks, cattle and humans. In this area, a recent survey showed a yearlong presence of JEV vectors feeding on pigs, chickens, humans, dogs and cattle [26,40]. Although JEV host densities are low (353 chickens and 66 pigs per km^2^ in Kandal province [37,41]), this multi-host system could allow for year-round circulation of JEV.

The overall exposure of the human population to JEV is probably underestimated and the clinical incidence of JE in Cambodia remains difficult to assess [1,7], although this would help to promote vaccination campaigns. Measuring the exposure of dogs to JEV could be an alternative solution. Indeed, exposure of dogs to JEV has been shown in Singapore and Japan (with JEV neutralizing antibodies detected in 17% to 39.6% of dogs sampled) [32,42]. In Kandal province, the dog-to-human ratio has been estimated to 1:3.8 in 2017 [43]. A recent serological survey carried out in the same area showed that 35% of sampled dogs had JEV neutralizing antibodies [16], and blood meal analysis of *Culex spp*. trapped in this area confirmed that JEV vectors can feed on dogs [26]. As dogs live in close proximity to humans, their exposure to JEV could be a good proxy for human exposure.

The objectives of this study were (i) to evaluate R_0_ and predict, based on simulations, human exposure to JEV in villages of Kandal, a rural province of Cambodia, (ii) to analyse how, in such villages, host community composition affects R_0_ value and human exposure, and (iii) to assess the potential use of dogs as sentinels of human exposure to JEV.

## Materials and methods

### Study area, host populations and serological data

The study focuses on three rural districts of Kandal province numbered 1–3 below (D1: Khsach Kandal, D2: Kien Svay and D3: Kaoh Thum), located northeast, east and southeast of Phnom Penh respectively. In these 3 districts, a JEV serological survey was carried out in 2018 to assess the relative exposure to JEV of pigs, ducks, chickens, and dogs [16]. During this survey, the number of animals and humans per household was noted for each backyard where animals were sampled. To calculate the average population size per animal species and per village in each of the three districts, we used the number of households per village provided by the communal offices of 9 of the 20 villages visited. According to these village authorities, on average, 20% of the households raised pigs and 90% raised chickens. The percentage of households breeding ducks (30%) was computed based on our field observations, by calculating a ratio of households breeding ducks to households breeding chickens (Table 1). In the three districts of the study area, 112 pigs, 185 chickens, 128 ducks and 188 dogs from 20 villages were blood-sampled between March and December 2018. Samples were analysed by hemagglutination inhibition and virus neutralization assays. Depending on the district, neutralizing antibodies were found in 0 to 42% of the pigs, 0 to 33% of the ducks, 0 to 0.02% of the chickens, and 35% of the dogs [16].

**Table 1:** Average host population sizes in a traditional village of the three studied districts.

District	Pigs	Ducks	Chicken	Dogs	Cattle	Humans
D1	516.4	2020	7330	232	53.3	1630
D2	3644	509	14766	1460	39.2	4062
D3	3275	683	10312	1203	156	3880

### Model description

#### Structure of the model

We developed a model of JEV transmission between competent hosts (pigs, ducks, and chickens) and vectors (*Culex spp*.), and from vectors to non-competent hosts (cattle, humans, and dogs) at the village scale. This model was built to allow computing R_0_ and to analyse the epidemiological equilibrium situation. For this reason, and since previous studies conducted in the study area had shown high abundance of JEV vectors [40,44,45] and an intense circulation of the virus (seroconversion before 4 months of age in sentinel piglets, on average) [44,45], we chose to implement the model as a deterministic system, using ordinary differential equations. A detailed model description is given in S1 File. The epidemiological system represented a rural village of Kandal province where swine, ducks, chickens, cattle, dogs and humans are living. Because of different life expectancy, swine were split into two compartments: fattening pigs and sows. In the study area, these hosts are exposed to bites from *Culex tritaeniorhynchus*, *Culex vishnui* and *Culex gelidus*, the most abundant vectors of JEV in this region, and the feeding preferences of these vectors are similar [26]. For this reason, and due to the small surface area of villages in the study area (3–4 km^2^) compared to the flight range of *Culex spp*.[46], these different vector species were represented in the model by a single population of *Culex spp*. We assumed that direct pig-to-pig transmission and disease-induced death were negligible [47].

In our study area, pigs are raised in open pens, and poultry are often free roaming in the gardens during the day and locked in pens at night. Mosquito nets are rarely used in animal pens. Based on this livestock organization, we assumed that all hosts, including humans, were homogeneously exposed to the vector population. For this reason, and because direct transmission was neglected, it was not necessary to structure the host population by households.

We assumed that there was little or no exchange of infected hosts or vectors between the modelled and neighboring villages. Movements of live animals between villages are indeed extremely rare [37,41]. Only pigs may sometimes be transported to a slaughterhouse in a nearby village where they are slaughtered within two days. We assumed constant host population sizes, with births offsetting deaths or slaughters. The model incorporated seasonal variations in vector population size according to sinusoidal dynamics. A parameter denoted *ψ* (with 0≤*ψ*≤1) represented the amplitude of these variations, relative to the yearly average value *N*_*v*_: vector population size could then vary between *N*_*v*_(1−*ψ*) in the middle of the dry season, and *N*_*v*_(1+*ψ*) in the middle of the rainy season, with *ψ* = 0 denoting a constant size of vector population throughout the year (see S1 File).

**Fig 1:**
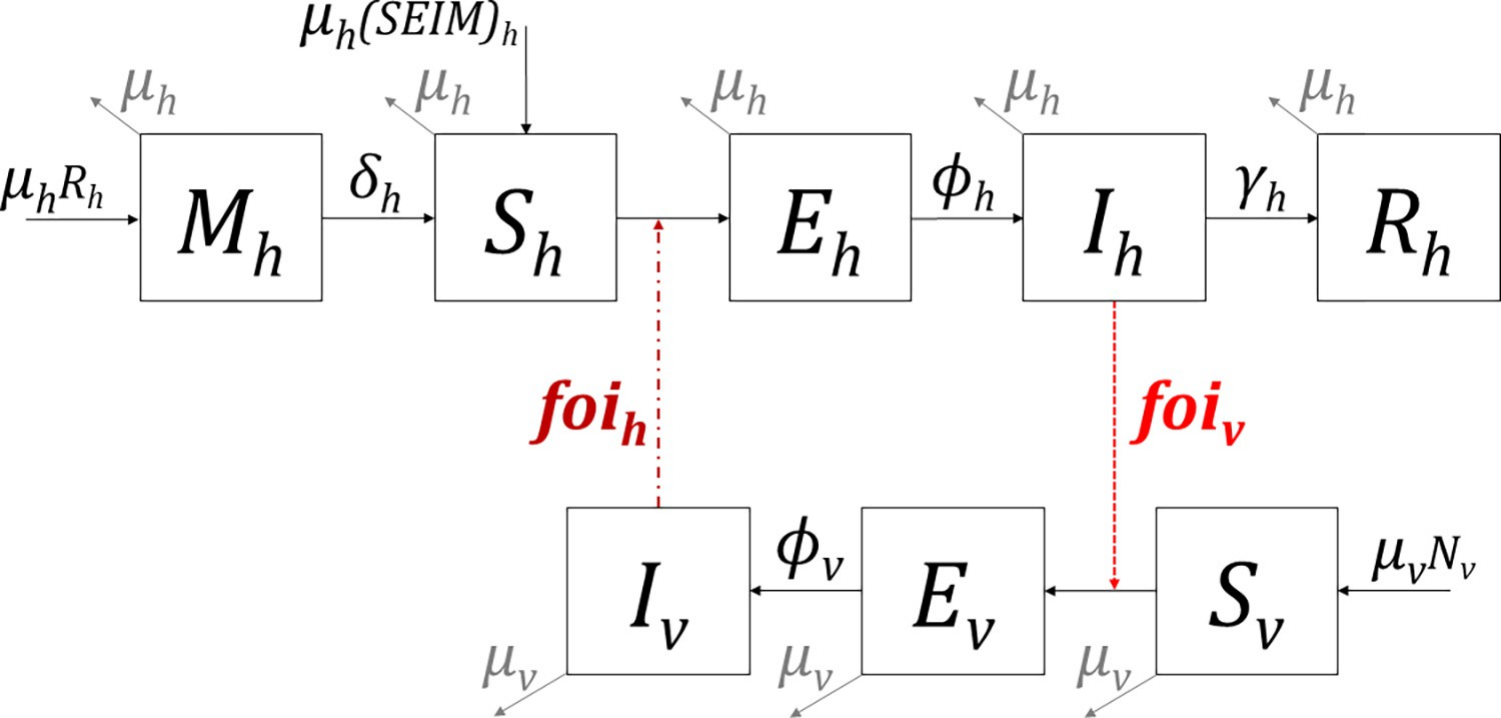
Schematic flowchart of the JEV model. The boxes represent the health states of hosts (h) and vectors (v): under maternal immunity (M), susceptible (S), latent (E), infected (I) and recovered (R). Solid-line arrows depict the flow into and out of compartments, determined by the associated parameters: **μ** = mortality rate, **δ** = 1/duration of maternal immunity, **Φ** = 1/duration of incubation period, **γ** = 1/duration of viremic period. N_v_ is the total population of vectors. Dashed arrows depict the forces of infection (foi) exerted by an infected vector on a susceptible host (foi_h_) and by infectious competent hosts on a susceptible vector (foi_v_). The force of infection exerted on a vector by a non-competent host is null as the probability of being infected (q) in this case is zero.

Fig 1 shows the flowchart of the model. Hosts were categorized into 5 infection states: protected by their maternal antibodies (*M*), susceptible (*S*), latent (*E*), infectious (*I*) and recovered(*R*). Hosts born from recovered females were assumed to be protected by maternal antibodies before becoming susceptible. After being bitten by an infectious mosquito, hosts had a species-specific probability (*p*) of entering the latent state (*E*). They then entered the infectious state after an incubation period (1/*Φ*_*h*_). After the infectious (viremic) period (1/*γ*_*h*_), hosts entered the recovered state (*R*) where they stayed until death (1/*μ*_*h*_). Although JEV vertical transmission has been documented in *Culex spp*., this phenomenon appears to be limited [48,49]. Moreover, the vector population and intensity of JEV transmission in the study area are such that we assumed this vertical transmission of JEV to be negligible: all vectors emerged in the susceptible state. Susceptible vectors had a given probability (*q*) of being infected and entering the latent state when biting an infectious host, *q* being null for non-competent hosts. After the extrinsic incubation period (of duration 1/*Φ*_*v*_), vectors became infectious and remained in the infectious state until death.

#### Forces of infection

The force of infection in hosts, *foi*_*i*_(*t*), is the instantaneous rate at time *t* that a susceptible host of species *i* becomes infected. It represents the transition rate between the susceptible state (S) and the latent state (E) and was defined in our model by:

$$foi_{i}(t)=bx_{i}p_{i}\frac{I_{v}(t)}{N_{i}}$$ (1)

where:

- *b* was the biting rate of the vector (number of bites per vector per day), or the inverse of the duration of its gonotrophic cycle,
- *p*_*i*_ was the transmission probability per bite from the vector to a host of species *i*,
- *I*_*v*_ (*t*) was the number of infected vectors at time *t*, and *N*_*i*_ the number of hosts of species *i*.
- *x*_*i*_ was the probability that a vector will choose a host of species *i* for its blood meal, calculated by multiplying the preference *π*_*i*_ for host *i* by the number of hosts of species *i*, divided by the sum of all preferences times host population sizes:
$$x_{i}=\frac{\pi_{i}N_{i}}{\sum_{j}\pi_{j}N_{j}}$$ (2)
  *x*_*i*_*p*_*i*_ was then the host-specific per bite transmission rate from the vector to host *i*, defined as the fraction of successful transmission events from one infected vector to a susceptible host of species *i*, per bite [50].

The global force of infection on vectors at time *t* was the sum of the forces exerted by the different host species at time *t*:

$$foi_{v}(t)=b\sum_{i}x_{i}q_{i}\frac{I_{i}(t)}{N_{i}}$$ (3)

Where *q*_*i*_ was the probability that a susceptible mosquito became infected after a blood meal taken on a viremic host of species *i*, the value of this parameter being > 0 for the competent host species (pigs, sows, ducks and chickens) and null for the non-competent host species (humans, dogs, cattle).

#### Parameterization

The definitions and values of the model parameters are provided in Table 2, and details of how fixed parameters were obtained are given in S1 File

**Table 2:** Parameters of the deterministic model.

Parameter	Definition	Value	References
	Hosts		
*1/μ*_*p*_, *1/μ*_*c*_	Average lifespan of pigs & chickens	6m*	Field data
*1/μ*_*s*_	Average lifespan of sows	3y	Field data
*1/μ*_*d*_	Average lifespan of ducks	2y	Field data
*1/μ*_*h*_	Average lifespan of humans	70y	[51]
*1/μ*_*b*_	Average lifespan of cattle	7y	Field data
*1/μ*_*dog*_	Average lifespan of dogs	5y	[43]
*1/δ*_*p*_	Duration of maternal immunity in pigs & sows	2.5m	[44,45]
*1/δ*_*d*_, *1/δ*_*c*_	Duration of maternal immunity in ducks & chickens	1m	[52,53]
*1/δ*_*h*_	Duration of maternal immunity in humans	5m	[54,55]
*1/δ*_*b*_, *1/δ*_*dog*_	Duration of maternal immunity in cattle & dogs	3m	[56,57]
*1/Φ*_*p*_	Incubation period in pigs & sows	2d	[58–60]
*1/Φ*_*d*_	Incubation period in ducks	2d	[19–22]
*1/Φ*_*c*_	Incubation period in chickens	1.5d	[19,61]
*1/Φ*_*h*_	Incubation period in humans	10d	[38]
*1/Φ*_*b*_, *1/Φ*_*dog*_	Incubation period in cattle & dogs	4d	[62,63]
*1/γ*_*p*_	Viremic period in pigs & sows	1.5d	[58,59]
*1/γ*_*d*_, *1/γ*_*c*_	Viremic period in ducks & chickens	3d	[19–21,61]
*1/γ*_*h*_, *1/γ*_*b*_, *1/γ*_*dog*_	Recovery period in humans & cattle & dogs	5d	[62–65]
	Vectors		
*1/μ*_*v*_	Average lifespan of *Cx*. *spp*.	25d	[66]
*b*	*Cx*. *spp*. biting rate	0.25	[67,68]
*1/Φ*_*v*_	Extrinsic incubation period in *Cx*. *spp*.	10d	[21,69]
*ψ*	Seasonal variations of vector population size	0	[40,44]
	Vector/Host interactions		
*π*_*p*_	Feeding preference of *Cx*. *spp*. for pigs & sows	1**	[26]
*π*_*d*_	Feeding preference of *Cx*. *spp*. for ducks**		Estimated
*π*_*c*_	Feeding preference of *Cx*. *spp*. for chickens**	0.09	[26]
*π*_*h*_	Feeding preference of *Cx*. *spp*. for humans**	0.5	[26]
*π*_*b*_	Feeding preference of *Cx*. *spp*. for cattle**	1.7	[26]
*π*_*dog*_	Feeding preference of *Cx*. *spp*. for dogs**		Estimated
*p*	Vector (*Cx*. *spp*.) to host transmission probability	0.5	[70,20]
*q*_*p*,_ *q*_*d*,_ *q*_*c*_	Competent host to vector (*Cx*. *spp*.) transmission probability	0.5	[21,69–72]
*q*_*b*_, *q*_*dog*_, *q*_*h*_,	Non-competent host to vector (*Cx*. *spp*.) transmission probability	0	[62,63]

Subscripts: p = pigs, s = sows, d = ducks, c = chickens, b = cattle, h = human.

* y = year; m = month; d = day.

** Feeding preferences relative to pig, used as a reference.

We estimated three of the model parameters which were unavailable in the literature, using seroprevalence data collected in the same area in pigs, poultry and dogs: the feeding preferences of *Culex spp*. for ducks (*π*_*d*_) and dogs (*π*_*dog*_), relative to pigs (used as a reference), as well as the number of vectors involved in JEV transmission. This latter parameter was separately estimated for each district (*N*_*v1*_, *N*_*v2*_, *N*_*v3*_) to control for differences between districts that would not be captured by the model, such as inter-district variations in vector breeding sites densities or in the accessibility of vectors to hosts. We used the Nelder-Mead optimization algorithm to minimize the negative log-likelihood of our serological data (number of positive among the tested animals in each host group and district), with respect to *N*_*v1*_, *N*_*v2*_, *N*_*v3*_, *π*_*d*_ and *π*_*d*_ (see S1 File for the definition of the likelihood function).

We used the “optim” function of R (version 4.0.2) [73] to minimize the log-likelihood. The variance-covariance matrix, obtained by inverting the Hessian matrix, was used to compute the confidence intervals of the estimated parameters.

### Model exploitation

#### Evaluation of R_0_ and human exposure in the study area

We first used the model to characterize the circulation of JEV in villages in each of the three study districts, simulating human exposure and evaluating the basic reproduction number (R_0_). The details of its computation are provided in S1 File. We then used three indicators computed on the 30^th^ simulated year to predict human exposure in the studied districts, assuming the epidemiological system was in a steady state:

- the annual probability of human exposure, which is the probability of receiving at least one infective bite over 1 year. This indicator was given by: $1-\exp\left(-\sum_{t=tmax-365}^{tmax}foi_{h}(t)\right)$, with *tmax* the last day of simulation (10,958^th^ day, see S1 File) and *foi*_*h*_(*t*) the value of the force of infection in humans at time *t*;
- the average age at infection, in years. This indicator was calculated as $1/\sum_{t=tmax-365}^{tmax}foi_{h}(t)$, assuming that people living in the modeled village are equally exposed to JEV since birth (this indicator was calculated only in situations where R_0_ was above 1);
- the annual incidence rate of human infections, which represents the proportion of persons infected in one year, at the epidemiological equilibrium. This indicator was given by $\frac{\sum_{t=tmax-365}^{tmax}foi_{h}(t)S_{h}(t)}{N_{h}}$, with *S*_*h*_*(t)* the number of susceptible people at day *t*, and *N*_*h*_ the total number or persons in the village.

The 95% confidence intervals (CIs) of R_0_ and of the three above indicators were calculated by drawing 1000 joint values of *N*_*v1*_, *N*_*v2*_, *N*_*v3*_, *π*_*d*_, and *π*_*dog*_, from a multidimensional Gaussian distribution centered on the estimated parameter values, and whose dispersion parameters were given by the variance-covariance matrix (produced by parameter estimation procedure). We ran the model using these 1000 joint values, computed R_0_ and human exposure indicators: CI bounds were the 2.5% and 97.5% percentiles of the resulting distribution. The model was implemented using R (version 4.0.2) [73].

#### Influence of host community composition on R_0_ and human exposure

In a second step, we used the model to analyse how the composition of the host community affected the ability of the epidemiological system to be invaded by JEV and influenced human exposure. To assess the effect of the host community composition variation on the estimated indicators, the size of the vector population had to remain constant in order to distinguish the effect of a change in host community composition from that of a change in the number of vectors. Second, variations in host community composition had to be realistic: since most animals are raised for self-consumption in the study area, the host community had to vary while maintaining a roughly constant level of food resources for villagers. For *Culicoides* midges, vector population size has been shown to depend on the total available body surface area (BSA) of hosts [74]. Assuming the same for *Culex spp*., varying the host community composition while keeping their total BSA equal to a reference value satisfied the first constraint. It also satisfied the second constraint, as BSA is linked to body mass by an allometric relationship. We thus varied the host community composition by changing the proportion of each host group in a constant total BSA. We analysed the effects on R_0_ and the three indicators of human exposure defined above (calculated from the simulations at epidemiological equilibrium), considering 3 types of variations of the host community composition:

- variation 1: relative share of competent hosts BSA, versus non-competent hosts BSA (percentage of competent hosts BSA ranging from 5% to 95% of the whole system BSA). As non-competent hosts “dilute” mosquito bites, we expected an impact on the proportion of infectious vectors;
- variation 2: relative share of pigs, chickens and ducks BSA among competent hosts BSA was also expected to influence JEV transmission dynamics, as each competent host has species-specific infection parameters (e.g. duration of viraemia), and vector feeding preferences differ from one species to another. For this variation, we fixed the percentage of chickens among poultry BSA at 5% (scenario A), 55% (scenario B) and 95% (scenario C) and made vary the proportion of pigs among competent hosts BSA.
- variation 3: relative share of cattle BSA among cattle-and-pigs BSA together (percentage of cattle BSA ranging from 0% to 100% of the cattle-and-pigs BSA). Cattle, being non-competent hosts on which *Culex spp*. feed, can dilute infecting bites and decrease R_0_. This variation was intended to explore this dilution effect on human exposure, the human population size being constant (contrary to variation 1).

For each of the three variations, the total reference BSA (held constant) was calculated based on the average number of hosts per category (Table 1) in villages of the 3 districts (using as weights the number of villages visited during the field survey): 3420 people, 2446 pigs, 285 sows, 941 ducks, 10639 chickens, 107 cattle and 1044 dogs. The BSA of a typical individual was calculated using existing allometric formulas [75–77]: 1.53 m^2^ for a pig, 3.47 m^2^ for a sow, 0.15 m^2^ for a duck, 0.13 m^2^ for a chicken, 1.81 m^2^ for a human, 4.29 m^2^ for a cattle, and 0.61 m^2^ for a dog.

The constant size of the vector population was the average of the three district-specific estimates of *N*_*v1*_, *N*_*v2*_ and *N*_*v3*_.

#### Use of dogs as sentinels of human exposure

Finally, we used the model to analyse the relationship between JE seroprevalence in dogs and the annual probability of exposure in humans. The relationship between dog seroprevalence and annual probability of human exposure may be influenced by (i) the proportion of each host species in the multi-host system (including the human/dog ratio), (ii) the average vector population size (*N*_*v*_), and (iii) the lifespan of dogs (linked to the mortality rate *μ*_*dog*_) as the probability of being seropositive increases with age in the case of an endemic disease and when antibodies remain all life long after a single infection as it is the case with Flaviruses. Therefore, we ran 10,000 simulations for which the composition of the host community, *N*_*v*_, and the lifespan of dogs varied randomly, and R_0_ was >1. For each simulation, the proportion of BSA of each host species was randomly drawn between 0.01 and 0.99 while ensuring that the overall BSA of the system remained constant. *N*_*v*_ was randomly drawn between 1,000 and 200,000 vectors, and the lifespan of the dogs was randomly drawn between 2 and 11 years, with their average observed lifespan being 5 years [43].

We then plotted the annual probability of human exposure to JEV as a function of JEV seroprevalence in dogs and visually analysed the relationship between these two indicators.

### Sensitivity analysis

To rank the model parameters according to their influence on R_0_, we first performed a semi-quantitative sensitivity analysis, using the Morris method [78]. Details are available in S2 File.

We then focused the sensitivity analysis on specific parameters, (i) for which hypotheses had been made when parameterizing the model, *i*.*e*. the seasonal variations of vector population size *N*_*v*_ (*ψ*), (ii) for which data available in literature were highly variable, *i*.*e*. the transmission probability from viremic hosts to vectors (*q*_*p*_, *q*_*d*_, *q*_*c*_), or (iii) for which the available data may not represent the field condition, *i*.*e*. the feeding preferences of *Culex spp*. for humans (*π*_*h*_). Following a “one at a time” plan and for the traditional villages of the three studied districts (Table 1) as well as for the three types of variations of the host community composition, we successively considered alternative values of these parameters. For each of these values, we re-estimated *N*_*v*1_, *N*_*v*2_, *N*_*v*3_, *π*_*duck*_, and *π*_*dog*_, computed R_0_ and the indicators of human exposure when the epidemiological system has reached a steady state. Two alternative values were considered for *q*_*p*_, *q*_*d*_, and *q*_*c*_: 90% lower and 90% higher. A decrease from 10% to 50% was considered for *π*_*h*_. Finally, and to be consistent with field observations [40,44], we considered a seasonal variation of the vector population size (*ψ*) of +/-20% relative to the average annual value.

## Results

### Parameter estimation and model fit

The estimated average size of the vector population was 17,789 mosquitoes for a traditional village of district D1 (*N*_*v1*_, 95% CI: 12,304–25,723), 52,353 mosquitoes for district D2 (*N*_*v2*_, 95% CI: 44,276–61,905), and 59,536 mosquitoes for district D3 (*N*_*v3*_, 95% CI: 49,993–70,902) (see Table 1 for average host population sizes in these villages). The estimated feeding preference of *Culex spp*. (relative to pigs that were used as reference) was 0.43 for ducks (*π*_*duck*_, 95% CI: 0.22–0.85), and 0.12 for dogs (*π*_*dog*_, 95% CI: 0.08–0.21). Model fit results based on observed and predicted JEV seroprevalences are reported in Table 3.

**Table 3:** Observed and predicted JEV seroprevalence per species in traditional villages of the three studied districts.

District	Species	Observed seroprevalence [16]		Predicted seroprevalence
**D1**	**Pigs**	0^a^/5^b^	0.00^c^ (0.00–0.52)^d^	0.05^c^ (0.0001–0.16)^d^
	**Ducks**	1/81	0.01 (0.00–0.07)	0.01 (0.00–0.05)
	**Chickens**	1/82	0.01 (0.00–0.07)	0.004 (0.00–0.01)
**D2**	**Pigs**	15/59	0.25 (0.15–0.38)	0.25 (0.15–0.36)
	**Ducks**	0/5	0.00 (0.00–0.52)	0.07 (0.03–0.14)
	**Chickens**	1/46	0.02 (0.00–0.12)	0.01 (0.007–0.02)
**D3**	**Pigs**	20/48	0.42 (0.28–0.57)	0.40 (0.27–0.52)
	**Ducks**	14/42	0.33 (0.20–0.50)	0.28 (0.18–0.40)
	**Chickens**	0/57	0.00 (0.00–0.06)	0.02 (0.02–0.04)
	**Dogs**	65/188	0.35 (0.28–0.42)	0.32 (0.26–0.38)

^a^Number of seropositive animals.

^b^Number of tested animals.

^c^Seroprevalence rate.

^d^95% confidence interval

### Model exploitation

#### Evaluation of R_0_ and human exposure in the study area

R_0_ was above 1 in the 3 districts (Table 4). The estimated annual probability of human exposure to JEV ranged from 9% to 47%, and the estimated incidence rate of human JEV infections ranged from 1.37% to 1.46%, corresponding to 24 to 56 people infected per year per village (Table 4, population sizes are given in Table 1). The estimated average age at infection varied between 2 years old and 11 years old.

**Table 4:** Estimated values of R_0_ and of human exposure indicators in traditional villages of the three studied districts.

District	R_0_ (95% CI)	Annual probability of human exposure to JEV (95% CI)	Annual incidence rate of human JEV infections (95% CI)	Annual incidence of human JEV infections (95% CI)	Average age at infection in humans (years) (95% CI)
**D1**	1.07 (0.996–1.20)	0.09 (0.0002–0.3)	0.0146 (0.0002–0.015)	23.7 (0.3–25.1)	10.6 (3.1- >100)
**D2**	1.25 (1.16–1.37)	0.34 (0.21–0.47)	0.0137 (0.0135–0.0139)	55.8 (54.9–56.4)	2.9 (2.0–4.6)
**D3**	1.38 (1.29–1.53)	0.47 (0.34–0.62)	0.0139 (0.0137–0.0140)	53.9 (53.3–54.3)	2.0 (1.5–2.8)

#### Influence of host community composition on R_0_ and human exposure

**Fig 2:**
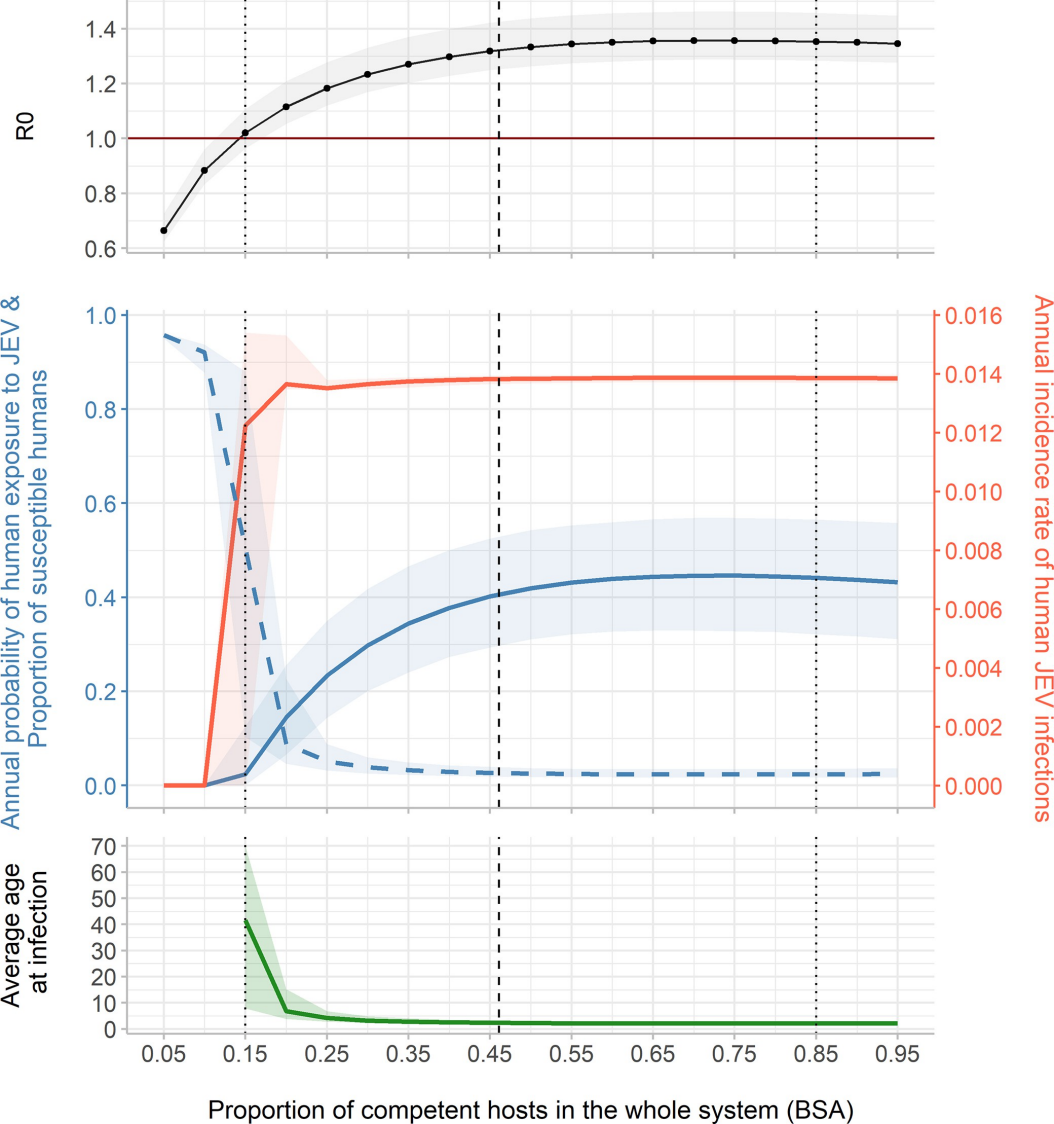
R_0_, annual probability of human exposure to JEV (blue solid line), proportion of susceptible humans (blue dashed line), annual incidence rate of human JEV infections (red line), and average age at infection, at the epidemiological equilibrium, according to the proportion of competent hosts in the whole system (BSA). The vertical dashed line corresponds to the host community composition observed in an average village of Kandal province. The two vertical dotted lines correspond to two contrasted host community compositions: one with 15% and the second with 85% of competent hosts BSA. Total BSA of hosts and the size of vector population (48,663 mosquitoes, 95% CI: 40,347–58,863) remain constant.

**Variation 1: Influence of the relative share of competent hosts BSA versus non-competent hosts BSA.** In an average village of Kandal province, competent hosts represented 46.1% of the total BSA of the village (Fig 2, dashed line). As *Culex spp*. had lower feeding preferences for competent hosts (pigs, poultry) than for cattle, which are non-competent hosts, a sufficient quantity of competent hosts was required to allow JEV to invade the epidemiological system. R_0_ became greater than 1 when the percentage of competent hosts BSA reached 15% of the whole system’s BSA. R_0_ then increased with the proportion of competent hosts and stabilized at about 1.35. When R_0_ was >1, the annual probability of human exposure to JEV at the equilibrium varied from 0.024 to 0.45 and the annual incidence rate of human JEV infections varied from 0.012 to 0.014.

We compared two contrasted host community compositions, highlighted in Fig 2 (dotted lines), the first with 15% of competent hosts BSA, and the second with 85% of competent hosts BSA. In the first host community composition (left dotted line), an individual had an annual probability of exposure to infection of 0.02. The predicted annual incidence rate of human infections was 0.0122, corresponding to 72.4 infections among 5939 people. In the second host community composition, the annual incidence rate of human infections was similar (0.0139), although the predicted number of infections was lower than in the first situation (13 infections) due to the lower population size (952 people). Conversely, the annual exposure probability was much higher in the second than in the first host community composition, *i*.*e*. 0.44.

Interestingly, the annual incidence rate of human infections was quite stable although the probability of exposure increased with the proportion of competent hosts in the system. Assuming homogeneous exposure of all age groups, the predicted average age at infection was 40 years old in the first host community composition (left dotted line) where the probability of exposure was low. In the second situation (right dotted line), where the probability of exposure was high, the predicted average age at infection was 2 years old. In the first host community composition, the annual incidence of infections was therefore distributed among all age groups, whereas in the second host community composition, infections were concentrated on the youngest age groups.

**Fig 3:**
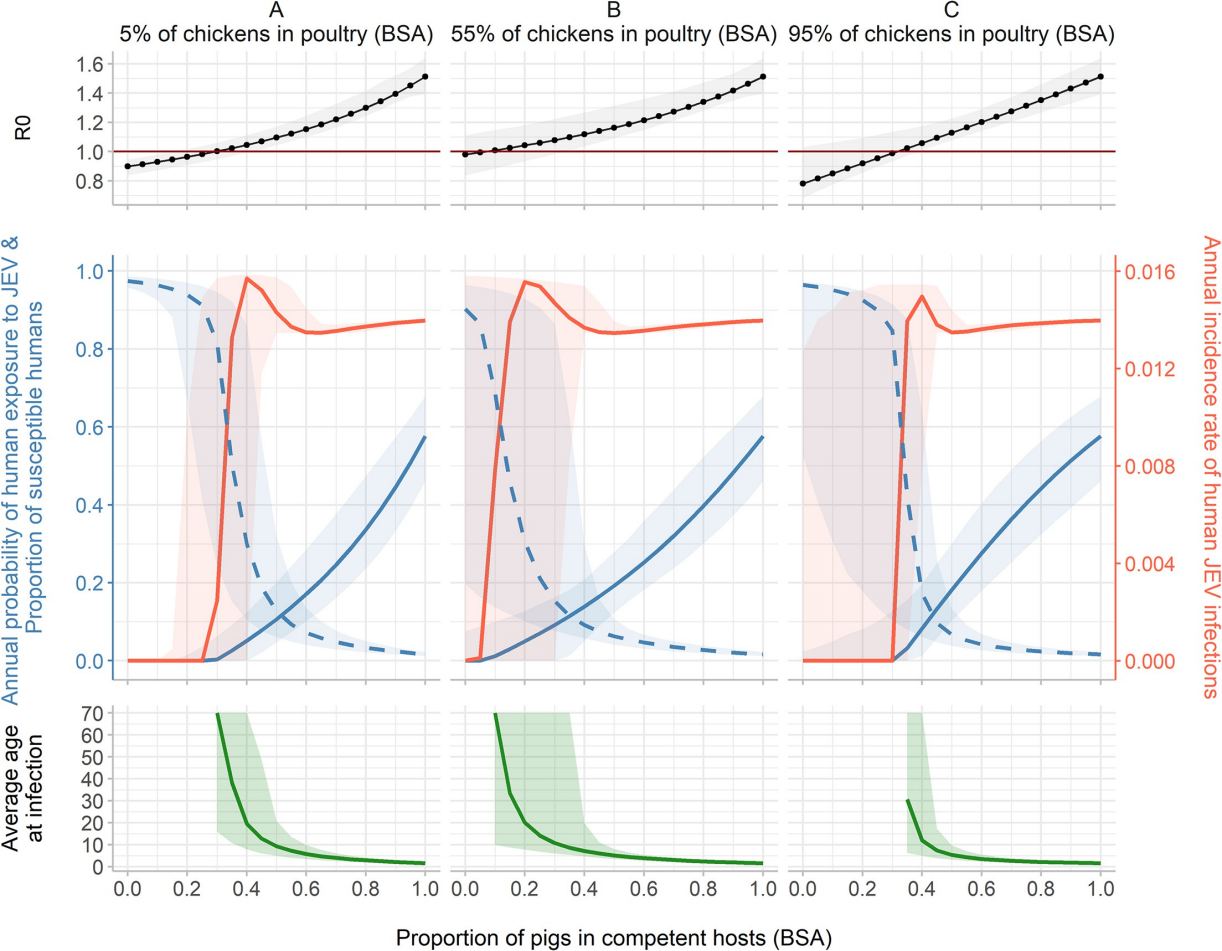
R_0_, annual probability of human exposure to JEV (blue solid line), proportion of susceptible humans (dashed blue line), annual incidence rate of human JEV infection (red line), and average age at infection, at the epidemiological equilibrium, according to the proportion of pigs in competent hosts (BSA), for three fixed percentages of chickens among poultry (BSA): 5% (scenario A), 55% (scenario B) and 95% (scenario C). Total BSA of hosts and the size of vector population (48,663 mosquitoes, 95% CI: 40,347–58,863) remain constant.

**Variation 2: Influence of the relative share of pigs, chickens and ducks BSA among competent hosts BSA.** In an average village of the study area, pigs BSA represented 35% of the whole village’s BSA and 76% of the competent hosts BSA. Chickens BSA represented 91% of the poultry (chickens and ducks) BSA (Fig 3).

Due to the strong feeding preference of vectors for pigs and sows (lower than for cattle but twice higher than for ducks and 10 times higher than for chicken, Table 2), R_0_ was mainly influenced by the proportion of pigs BSA, and it increased almost linearly with this proportion, regardless of the percentage of chickens among poultry BSA. In scenario A (percentage of chicken in poultry BSA set to 5%), R_0_ was >1 when there was more than 30% of pigs in competent hosts BSA. In scenario B (percentage of chicken in poultry BSA set to 55%), R_0_ was > 1 with only 10% of pigs in competent hosts BSA. At first, R_0_ slightly increased also with the percentage of chickens in poultry BSA because their lifespan (6 months) is shorter than that of ducks (2 years): a short lifespan ensures a faster turnover of susceptible animals in the system. However, when the percentage of chicken among competent hosts was very high (95%, scenario C), R_0_ became >1 with at least 35% of pigs in competent hosts BSA. Indeed, the mosquitoes’ feeding preference for chickens is the lowest among the six studied species (*π*_*c*_ = 0.09). When there were almost only chickens among the competent hosts, vectors bit the non-competent hosts more frequently, resulting in a decrease of R_0_.

Starting from 25% of chickens in poultry BSA (S1 Fig), and with 55% of chickens in poultry BSA (Fig 3, scenario B), and no pigs, R_0_ was close to 1 and its 95% confidence interval encompassed 1.

When R_0_ was >1, the annual probability of human exposure to JEV at the equilibrium varied from 0.004 to 0.58 and the annual incidence rate of human JEV infections varied from 0.003 to 0.015. In scenario B, 20% of pigs in competent hosts BSA led to a predicted annual probability of exposure of 0.05 and a predicted annual incidence rate of 0.0155 (corresponding to 53.2 infections per year per village). In the same scenario but with 80% of pigs in competent hosts BSA, the annual exposure probability reached 0.40, and the annual incidence rate was similar: 0.0138 (corresponding to 47.2 infections per year per village).

Again, the annual incidence rate of infection varied slightly whereas the annual probability of exposure increased with the proportion of pigs in the competent hosts BSA. As the latter increased, the average age at infection decreased and infections were more likely concentrated in the younger age groups. In scenario B, the average age at infection was over 70 years old (the graphs have been truncated above the lifespan of humans), over 20 years old and less than 5 years old with respectively 10%, 20% and more than 50% of pigs in competent hosts BSA.

**Fig 4:**
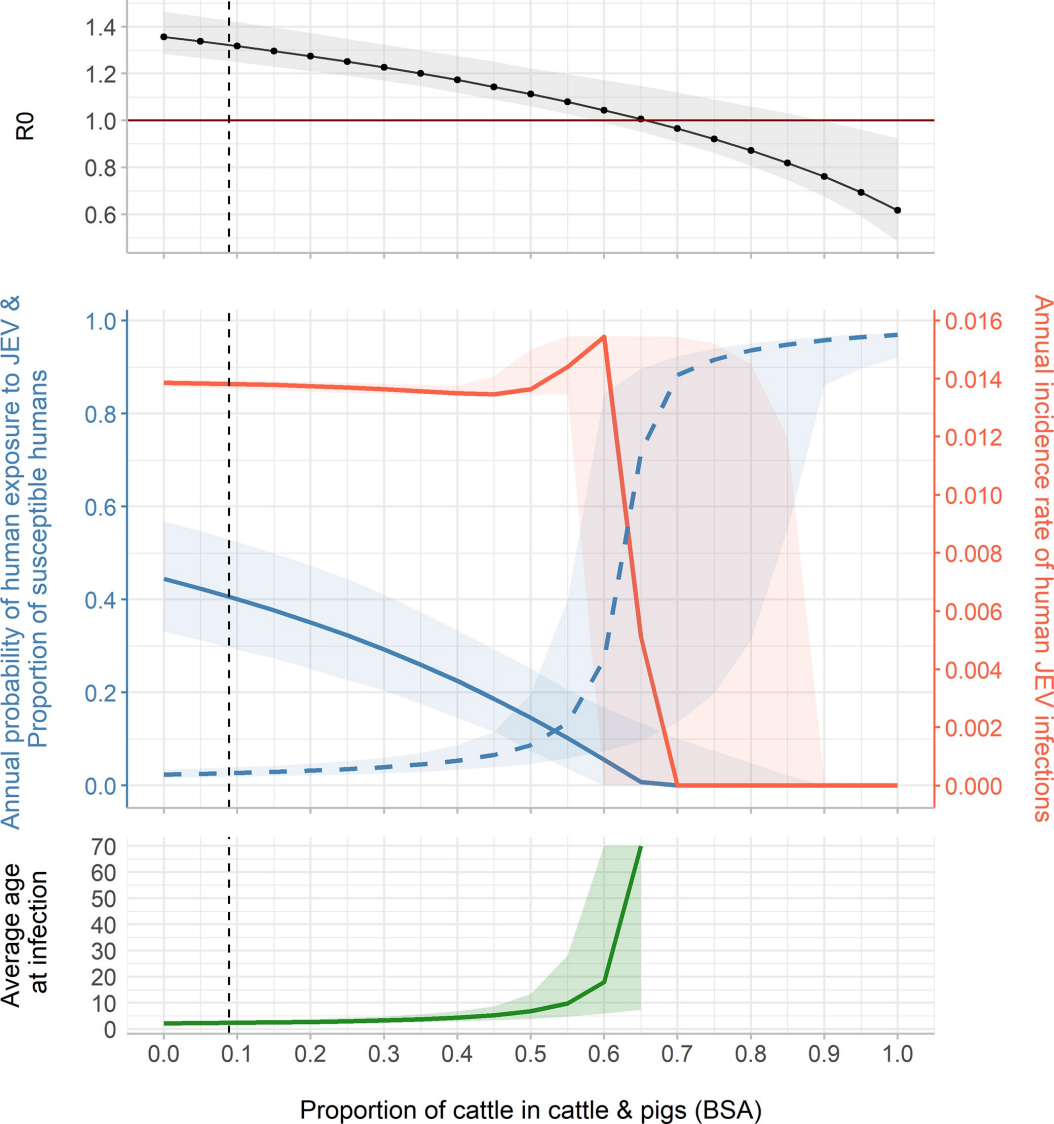
R_0_, annual probability of human exposure to JEV (blue solid line), proportion of susceptible humans (blue dashed line), annual incidence rate of human JEV infections (red line), and average age at infection, at the epidemiological equilibrium, according to the proportion of cattle among cattle and pigs (BSA). The vertical dashed line corresponds to the host community composition observed in an average village of Kandal province. Total BSA of hosts and the size of vector population (48,663 mosquitoes, 95% CI: 40,347–58,863) remain constant.

**Variation 3: Influence of the relative share of cattle BSA among cattle-and-pigs BSA.** In an average village of Kandal province, cattle BSA represented 8.9% of the total cattle-and-pigs BSA. Due to the strong feeding preference of vectors for cattle (the highest of all competent and non-competent hosts, Table 2), R_0_ and human exposure decreased as the percentage of cattle increased (Fig 4). From 65% of cattle among the total cattle-and-pigs BSA, R_0_ fell below 1. The annual incidence rate was maximal, *i*.*e*. 0.015, when there was 60% of cattle among cattle-and-pigs BSA (corresponding to 23% of cattle and 15% of pigs in the whole system’s BSA). This incidence rate corresponded to 51.3 human infections per year in the village (3420 people). For this system composition, the annual probability of human exposure to JEV was 0.06 and the average age at infection was approximately 18 years old.

#### Use of dogs as sentinels of human exposure to JEV

**Fig 5:**
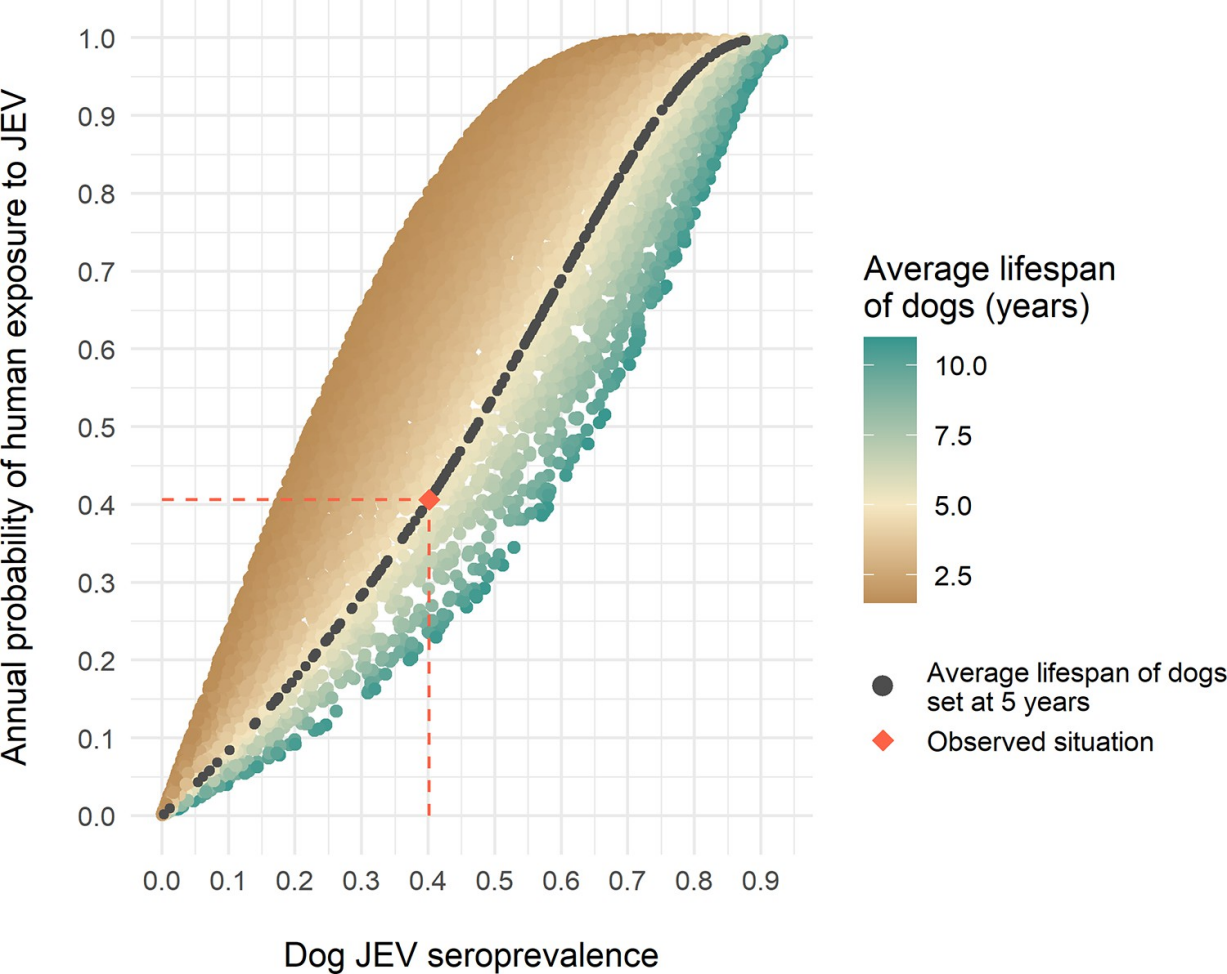
Annual probability of human exposure to JEV according to dog JEV seroprevalence.

The relationship between the predicted annual probability of human exposure and the predicted seroprevalence in dogs appeared roughly linear (Fig 5), with the slope of the curves influenced only by the average lifespan of dogs (S2 Fig). It is worth noting that, for the dog average lifespan estimated in the study area (5 years [43]), the slope of the curve was close to 1, the annual probability of human exposure to infective bites being then approximately equal to the seroprevalence in dog. This result suggests that, in the studied area, JE seroprevalence in dogs would be a good proxy for human exposure to JEV infection.

### Sensitivity analysis

Details of Morris sensitivity analysis results are available in S2 File. Among the 51 model parameters, 9 had a significant and linear influence on R_0_. As expected, these were mostly vector and pig related parameters: the vector biting rate *b*, the vector mortality rate *μ*_*v*_, the average vector population size *N*_*v*_, the pig recovery rate *γ*_*p*_, the probability of JEV transmission from pigs to vectors *q*_*p*_, the probability of JEV transmission from vectors to pigs *p*_*p*_, and the feeding preference of vectors for pigs *π*_*p*_.

Details of sensitivity analysis of model parameter estimates, R_0_ and human exposure indicators to host-to-vector transmission probability and to *Culex spp*. feeding preference for humans, in traditional villages of the three studied districts, are given in S2 File. Large variations in the value of host-to-vector transmission probability had almost no impact on R_0_ and on human exposure indicators in the host community composition observed in the field (Table A in S2 File). A 50% decrease in the vector feeding preference for humans, based on the assumption that humans may be less exposed to mosquito bites than animals living outdoors, did not influence parameter estimates, but resulted in a doubling of the average age of infection in district D1 (Table B in S2 File). Finally, incorporating seasonal variations of vector population size did not significantly influence R_0_ and human exposure indicators (Table C in S2 File).

The indicators (R_0_, annual exposure probability, annual incidence rate and average age at infection of humans) were then calculated for each variation in host community composition with the new input parameter values and parameter estimates (S2 File). In variation 1 (relative share of competent hosts versus non-competent hosts BSA), varying input parameters had almost no impact on output indicators, except an expected decrease of human exposure probability when *π*_*h*_ was halved. Overall, the main qualitative results obtained previously, *i*.*e*. an R_0_ increasing with the proportion of competent hosts and a distribution of annual incidence of infections concentrated on the youngest age groups when there is a majority of competent hosts in the system, were not changed (Fig B in S2 File). In variation 2 (relative share of pigs, chickens and ducks among competent hosts), the 90% decrease in *q*_*p*_ led to a profound change in the dynamics of the system since, on the one hand, the estimated averaged *N*_*v*_ was much larger than in the initial situation, and on the other hand, the system converged towards a transmission dynamic where the virus circulated mainly between birds. When modifying the other input parameters (*q*_*d*_, *q*_*c*_, *π*_*h*_, *ψ*), key qualitative results were not changed compared to those obtained from simulations with the default parameters (Fig C in S2 File). In variation 3 (relative share of cattle among cattle and pigs), varying input parameters had almost no impact on output indicators and key qualitative results presented above remained similar with those obtained from simulations performed with the default parameters (Fig D in S2 File).

## Discussion

Our model, calibrated on field-collected serological and demographic data first showed an intense circulation of JEV in Kandal province, with R_0_ values ranging from 1.07 and 1.38 and an annual probability of human exposure from 9 to 47% depending on the district. The average age at infection was always low, *i*.e. between 2 and 11 years old suggesting an important clinical impact on children health in absence of vaccination. Secondly, the simulation results confirm previous experimental results [19–24]: poultry could serve as a reservoir and JEV could invade a system without pigs. Lastly, dogs might be a good proxy for human JEV exposure in the study area.

Estimation of the model parameters was based on the assumption that the system in which samples were collected to calculate seroprevalences was at endemic equilibrium state, which may not be accurate. However, serological studies performed in the same region suggest little seasonal and inter-annual variation in JEV circulation in pigs [44,45] and ducks [8,16], and allowed us to make this assumption.

Available data on the clinical incidence of JEV in Cambodia are scarce and come mainly from hospital-based studies, sometimes in areas where access to care structures and clinical case identification capacities are limited [33,36,79,80]. The community-level clinical JEV incidence is thus probably underestimated. Under these conditions, Tarantola et al. (2014) estimated that 1/250 to 1/500 JEV infections resulted in symptomatic cases in Cambodia [81]. Based on these ratios and the annual incidence of human JEV infections predicted by our model (between 23.7 and 56 infections per year in a village of 1630 and 4062 people respectively), one can expect 0.05 (if the rate is 1/500) to 0.09 (if the rate is 1/250) clinical cases per year per village in district D1, and 0.11 to 0.22 clinical cases per year per village in districts D2 and D3. Although this is only a rough estimate, it would correspond to a clinical incidence of 2.8 to 5.8/100,000 JEV cases per year. In comparison, Mao et al. (2020), and Tian et al. (2015), estimated the average annual incidence of JEV cases in Yunnan and Changsha provinces, China, to be 0.16/100,000 (in 2017, after 10 years of vaccination program) and 0.15/100,000 respectively, by collecting hospital data for 6 to 10 years [82,83]. After 19 years of follow-up, Montini et al. (2020) estimated an incidence of 0.16/100,000 cases per year in Malaysia [84]. In Bhutan, 0.3/100,000 and 0.8/100,000 cases per year were estimated in 2020 for adults and children respectively, based on data from 5 sentinel hospitals [85]. Finally, Campbell et al. (2011) extrapolated hospital-based data of 12 southeast Asia countries, and estimated a global incidence of 1.8/100,000 and 5.4/100,000 cases per year for adults and children respectively, in all the 24 countries where JEV circulates [7]. Although they only concern the province of Kandal in Cambodia, our estimates are consistent with the latter figures. However, these exposure figures are to be set against the JE vaccination data in Cambodia since the consequence of this exposure will depend on the vaccination coverage of the population. The implementation of vaccination in Cambodia is recent, and the reported number of individuals vaccinated went from 0 before 2010 to more than 500,000 in 2015 [1]. Even if Quan et al, 2020 estimated a consequent reduction of JE cases through vaccination [1], JEV was still reported in 2017, after the main vaccination campaigns in 2016, as the primary cause of acute meningitis-encephalitis in children, with 35% of 1160 patients confirmed or highly probable to have JEV infection [36].

The proportion of severe cases occurring in Kandal province remains unknown. However, the predicted average age at infection in the modeled villages was very low, (ranging from 2 to 11 years old). Since young children are more likely to develop severe forms after JEV infection, as well as severe sequelae [2,33,82,84,85], these results and the current knowledge we have about JEV circulation in Cambodia would justify to intensify child immunization.

According to our results, variations of host community composition would influence the average age at infection: in peri-urban areas (with few competent hosts in the system (Fig 2) or few pigs among competent hosts (Fig 3)), infections might be concentrated in adults, whereas in rural areas (with more competent hosts in the system (Fig 2) or more pigs among competent hosts (Fig 3)), infections might be concentrated in the younger age groups. In the three studied districts, the modelled villages belonged to the latter category where the predicted average age of infection was low while the exposure probability was high.

Results of simulations confirmed the major role played by pigs in JEV circulation. However, it appeared that in a system without pigs, the upper bound of R_0_ confidence interval remained >1 when there was more than 25% of chickens in poultry BSA (S1 Fig). This suggests that, in a pig-free system, chickens and ducks could be sufficient for the virus to invade the epidemiological system. As shown by the sensitivity analysis, our results appeared robust with respect to the values of uncertain parameters (*q*_*p*_, *q*_*d*_, *q*_*c*_, *π*_*h*_) or for which specific assumptions had been made during model parameterization.

As Lord et al. (2015) and Bae et al. (2018) have already pointed it out, the epidemiology of JEV needs to be rethought, as JEV circulation could be maintained, or not, within complex and area-dependent epidemiological systems [13,14].

Epidemiological systems are bound to change, for political, economic, cultural or sanitary reasons. Traditional pig farming has been widespread in Cambodia since before the 1950s. The Khmer Rouge period constituted a rupture in the country’s history, and pig farming resumed only from the 1980s [37,41]. Since then, traditional pig farming has been facing sanitary crises such as Classical Swine fever and recently African Swine fever (ASF) [86], which could drastically reduce swine density. Moreover, the industrialization of the sector and the competition with intensive production, notably from Thailand, is driving down the price of meat and gradually discouraging small farmers for whom traditional pig farming is no longer profitable (National Animal Health and Production Research Institute, personal communication). On the other hand, landscape management, through drainage or reduction of rice fields due to industrialization, as well as climate change and climatic hazards could also transform the epidemiological system by directly affecting host and vector populations, and thus the transmission pattern of JEV. The changes in host community composition we have simulated are thus likely to occur and the current sanitary context related to the circulation of ASF in Asia is a concrete example of these changes. In Vietnam, more than 21% of the total pig herd was decimated in 2020 due to the ASF outbreak. The reduction of the total number of pig herds has led to a rapid growth of cattle production (+5% in one year) [87]. Our model confirmed that R_0_ might decrease and the level of virus circulation at endemic equilibrium be lower in systems where the proportion of cattle increases. This zooprophylactic-like effect is explained by the fact that cows are non-competent hosts on which JEV vectors feed [26,28].

In our study area, it has been shown that dogs, which are numerous and live in close proximity to humans, are widely exposed to JEV infection [16]. In an endemic area where dogs live an average of 5 years, the annual probability of human exposure to JEV was similar to the value of seroprevalence in dogs (Fig 5). The simulations indicated that the relationship between these two indicators may be generalizable, as it was approximately linear regardless of the size and composition of the host community and the size of the vector population (Fig 5). In the particular context of Kandal province, seroprevalence in dogs may be a good proxy for human exposure, and a tool for estimating the impact of JEV on public health. If this result was confirmed in varied epidemiological contexts, the practical use of dogs as sentinels for human JEV exposure would depend on the epidemiological situation of the region. In endemic contexts as in Cambodia, a verified correlation between dog seroprevalence and human exposure probability would help quantifying people exposure, by implementing serological surveys in dogs. In epidemic contexts where JEV circulation is seasonal as in Thailand or Vietnam [39,88], detecting JEV circulation in sentinel dogs would help implementing prevention or information measures ahead of the expected waves of exposure. In disease-free areas, sentinel dogs could be used as an early-surveillance system of JEV emergence in risky areas. Exposure data from sentinel dogs can also be used to target vaccination to areas where expected human exposure is greatest or where access to JEV vaccine or resources to implement vaccination are limited. Even if in-depth surveys under various environmental conditions should be further implemented to infer the potential use of dogs as JEV sentinels in the future, this complements the results of studies suggesting that dogs could be used as sentinels for other flaviviruses such as WNV [89,90].

Our theoretical approach showed that variations of the composition of the multi-host system identified in Cambodia may have an impact on the ability of the epidemiological system to sustain JEV transmission, on the human exposure to JEV, and thus on the disease burden in humans, especially in young children. Besides children vaccination in JEV endemic areas, a proper evaluation of the impact on human health is needed as well as further investigation on the potential use of dog as sentinels of human exposure, to target prevention actions and reduce JEV burden in Cambodia.

## Supporting information

S1 File Equations of the deterministic compartmental model (I), Model parametrization and likelihood function (II) and Next Generation Matrix method for R0 computation (III). (DOCX)

S2 File Morris sensitivity analysis of R0 to parameter values (I), sensitivity analysis of model parameter estimates, R0 and human exposure indicators to host-to-vector transmission probability (*q_p_*, *q_d_*, *q_c_*), to *Culex spp*. feeding preference for humans (*π_h_*), and to +/-20% seasonal variations of vector population size (*ψ* = 0.2) in traditional villages of the three studied districts (II) and sensitivity analysis of R0 and human exposure indicators to *q_p_*, *q_d_*, *q_c_*, *π_h_*, and *ψ*, for the 3 variations of host community composition (III). (DOCX)

S1 Fig Variations of R_0_ according to the proportion of chickens among poultry (BSA), and for five percentages of pigs among competent hosts (BSA). The black point corresponds to the R_0_ for 100% of pigs in competent hosts BSA.
(TIF)

S2 Fig Annual probability of human exposure to JEV according to dog JEV seroprevalence, for varying human-dog ratios and vector population sizes. (TIF)
