# Supplementary material for: Modelling Japanese encephalitis virus transmission dynamics and human exposure in a Cambodian rural multi-host system
Source: PLoS Negl Trop Dis. 2022 Jul 11;16(7):e0010572. doi: 10.1371/journal.pntd.0010572 (PMC9302853; doi:10.1371/journal.pntd.0010572)
Supplement: S1 File — (DOCX) [file pntd.0010572.s001.docx]

**S1 File.** **Equations of the deterministic compartmental model (I), Model parametrization and likelihood function (II) and Next Generation Matrix method for R_0_ computation (III).**

1. Equations of the deterministic compartmental model

Seven host populations (pigs, sows, ducks, chickens, cattle, humans, dogs) are represented by the same system of equations, where the subscript *i* refers to the species. Five health states are distinguished: *M* (hosts protected by their maternal antibodies), *S* (susceptible hosts), *E* (latent hosts), *I* (infectious hosts) and *R* (recovered hosts). *Nv* is the number of vectors (*Cx. spp*) involved in JEV transmission and *Ni* the number of hosts *i* in the modeled village.

$\left\{ \begin{matrix} \frac{\begin{aligned} dM_{i} \end{aligned}}{dt}=\mu_{i}R_{i}-\delta_{i}M_{i}-\mu_{i}M_{i} \\ \frac{dS_{i}}{dt}=\delta_{i}M_{i}+\mu_{i}\left( S_{i}E_{i}I_{i}M_{i} \right)-\left( \frac{bx_{i}p_{i}I_{v}}{N_{i}} \right)S_{i}-\mu_{i}S_{i} \\ \begin{matrix} \begin{matrix} \frac{dE_{i}}{dt}=\left( \frac{bx_{i}p_{i}I_{v}}{N_{i}} \right)S_{i}-\phi_{i}E_{i}-\mu_{i}E_{i} \end{matrix} \\ \begin{matrix} \frac{dI_{i}}{dt}=\phi_{i}E_{i}-\gamma_{i}I_{i}-\mu_{i}I_{i} \\ \frac{dR_{i}}{dt}=\gamma_{i}I_{i}-\mu_{i}R_{i} \end{matrix} \end{matrix} \end{matrix} \right.$

$\mathrm{with} x_{i}= \frac{\pi_{i}N_{i}}{\sum_{j} \pi_{j}N_{j}}$

One vector population (of *Culex spp*) is represented by the following system of equations, where the health state *S_v_* corresponds to uninfected vectors, *E_v_* to latent vectors, and *I_v_* to infectious vectors. $foi_{v}$ is the force of infection exerted on vectors.

$$\left\{ \begin{matrix} \frac{dS_{v}}{dt}=\mu_{v}N_{v}(1+\psi\times sin(\frac{2\pi t}{365}+\frac{\pi}{2})-foi_{v}S_{v}-\mu_{v}S_{v} \\ \begin{matrix} \begin{matrix} \frac{dE_{v}}{dt}=foi_{v}S_{v} \end{matrix}-\phi_{v}E_{v}-\mu_{v}E_{v} \\ \begin{matrix} \frac{dI_{v}}{dt}=\phi_{v}E_{v}-\mu_{v}I_{v} \end{matrix} \end{matrix} \end{matrix} \right. \mathrm{with} foi_{v}=b\sum_{i} x_{i}q_{i}\frac{I_{i}}{N_{i}}$$

**Table A: Model parameters notations and meanings**

| **Parameter** | **Definition** |
| --- | --- |
| **Hosts** | |
| *1/μ_i_* | Average lifespan of host *i* |
| *1/δ_i_* | Duration of maternal immunity in host *i* |
| *1/Φ_i_* | Incubation period in host *i* |
| *1/γ_i_* | Viremic period in host *i* |
| **Vector** | |
| *1/μ_v_* | Average lifespan of *Cx. spp* |
| *b* | *Cx. spp* biting rate |
| *1/Φ_v_* | Extrinsic incubation period in *Cx. spp* |
| *ψ* | Seasonal variations of vector population size |
| **Vector/Hosts interactions** | |
| *π_i_* | Feeding preference of *Cx. spp* for host *i* |
| *p_i_* | Vector (*Cx. spp*) to host *i* transmission probability |
| *q_i_* | Host *i* to vector (*Cx. spp*) transmission probability |

1. Model parametrization and likelihood function
2. *Model fixed parameters*

We used field data collected during a serological survey led in 2018 [1] to determine the average lifespan of each host species (1/*μ*) in the modeled village. The respective durations of maternal immunity (1/*δ*), incubation (1/*Φ*), and recovery (1/*γ*) periods, the biting rate (*b*), as well as the vector-to-host transmission probability (*p*) were set according to the literature.

Estimates of host-feeding preference parameters (*π*) were obtained from a field study conducted by Boyer et al. (2021) in the study area in 2017 [2]. In this study, mosquitoes were captured using baited traps, in which individuals of four species (human, pig, cow, chicken) were exposed to mosquitoes. However, one could expect that humans, living indoors and possibly using mosquito nets, would be less exposed to mosquito bites. The feeding preference of *Culex spp.* for humans estimated by Boyer et al. may therefore overestimate its actual value under field conditions. The impact of this possible overestimation on our results was analysed in a sensitivity analysis (see the detailed methodology in main text, section “Sensitivity analysis”). Values of *π* for ducks and dogs were estimated by fitting the model as described in main text, section “Parametrization”.

Experimental studies of the transmission of JEV from competent hosts (*i.e.* pigs, chickens and ducks) to *Culex spp.* have reported large variations of the percentage of vectors to which JEV is transmitted, ranging from 0 to 100% [3–7]. Besides, the level of JEV viremia observed in pigs, ducks, and chickens vary substantially and might influence mosquito infection probability [8–10]. For these reasons, we first set the corresponding parameters (*q_p_*, *q_d_* and *q_c_*) to an intermediate value of 0.5 (main text, Table 2), and then made vary these values for each host to quantify the effect of changing these on model outputs (see “Sensitivity analysis” section in main text).

In the study area, field surveys have shown seasonal variations of the vector population size: Cappelle et al. [11] observed average numbers of vectors caught per night varying from -10% in the dry season to +10% in the wet season, compared to yearly average number of vectors caught per night. Similarly, Boyer et al. [12] observed variations of +/-30% around the mean value of the number of JEV vectors caught per night. However, epidemiological studies conducted in the study area suggested that, because of the high intensity of virus circulation in the area, these small amplitude variations have little impact on the force of infection exerted on hosts: the seroconversion rates of piglets from two cohorts followed by Cappelle et al. (2016) in Kandal province, one in the dry season and the other in the rainy season, were comparable [11]. For this reason, we first made the simplifying assumption that the value of parameter *ψ* was zero in our study area. Then, the influence of this hypothesis assumption on our results was evaluated in the sensitivity analysis.

1. *Likelihood function for model parameter estimation*

The likelihood ($L$) function was based on the observed seroprevalence data (number of positive among the tested animals in each host group and district), conditional to the force of infection predicted by the model for each sampled animal, on the calendar date the samples was taken, and assuming that the epidemiological system was at steady state (as JEV is considered endemic in the study area): $L=\prod_{v=1}^{3} L_{v}$, where $L_{v}$ was the likelihood of serological results obtained from samples taken in village *v*. This village-specific likelihood was: $L_{v}=\prod_{j=1}^{J_{v}} Q_{j} P_{j}+(1-Q_{j})(1-P_{j})$, where $J_{v}$ was the number of blood samples taken in village *v*, $Q_{j}=1$ if animal *j* was seropositive and 0 otherwise, and $P_{j}$ was the probability that the serum taken from animal *j* was seropositive, conditional to model predictions. Finally, this latter probability was: $P_{j}=1-\exp\left( -\sum_{t=T_{j}-{age}_{j}}^{T_{j}} {foi}_{i}\left( t \right) \right)$ where $T_{j}$ was the calendar date at which the sample was taken from animal *j*, ${age}_{j}$ its age (in days), and ${foi}_{i}\left( t \right)$ the force of infection exerted on animal *j* of species *i* on day *t*, as defined in eq. (1) (main text).

To ensure the steady state had been reached, we tested several simulation durations up to 200 years and set the simulation duration as the one from which the calculated human exposure indicators no longer varied: *tmax* = 10,958 days (30 years). The virus circulation in the system was initialized at the beginning of each simulation with the introduction of 100 infectious mosquitoes.

1. Next Generation Matrix method for R_0_ computation

R_0_ was computed from the model parameters using the Next Generation Matrix (NGM) method. The NGM describes the transmissions of a pathogen between the states-at-infection, being a combination of Jacobian matrices of transmission (rate at which new infections arise) and migration (rate at which individuals enter or leave the infectious compartment by other means) vectors. R_0_ is the dominant eigenvalue of NGM and describes the growth, in terms of generations, of the population of infected hosts [13].

1. We first isolated the compartments with infected (*E* and *I*) hosts and vectors, from our model’s ordinary differential equations:

$$\left\{ \begin{matrix} \frac{dE_{p}}{dt}=b x_{p} p_{p}\frac{I_{v}}{N_{p}}S_{p}- \varphi_{p}E_{p}-\mu_{p}E_{p} \\ \frac{dI_{p}}{dt}= \varphi_{p}E_{p}-\gamma_{p}I_{p}-\mu_{p}I_{p} \end{matrix} \right.$$

$$\left\{ \begin{matrix} \frac{dE_{s}}{dt}=b x_{s} p_{p}\frac{I_{v}}{N_{s}}S_{s}- \varphi_{p}E_{s}-\mu_{s}E_{s} \\ \frac{dI_{s}}{dt}= \varphi_{p}E_{s}-\gamma_{p}I_{s}-\mu_{s}I_{s} \end{matrix} \right.$$

$$\left\{ \begin{matrix} \frac{dE_{d}}{dt}=b x_{d} p_{d}\frac{I_{v}}{N_{d}}S_{d}- \varphi_{d}E_{d}-\mu_{d}E_{d} \\ \frac{dI_{d}}{dt}= \varphi_{d}E_{d}-\gamma_{d}I_{d}-\mu_{d}I_{d} \end{matrix} \right.$$

$$\left\{ \begin{matrix} \frac{dE_{c}}{dt}=b x_{c} p_{c}\frac{I_{v}}{N_{c}}S_{c}- \varphi_{c}E_{c}-\mu_{c}E_{c} \\ \frac{dI_{c}}{dt}= \varphi_{c}E_{c}-\gamma_{c}I_{c}-\mu_{c}I_{c} \end{matrix} \right.$$

$$\left\{ \begin{matrix} \frac{dE_{v}}{dt}=b(x_{p} q_{p}\frac{I_{p}}{N_{p}}+x_{s} q_{p}\frac{I_{s}}{N_{s}}+x_{d} q_{d}\frac{I_{d}}{N_{d}}+x_{c} q_{c}\frac{I_{c}}{N_{c}}+x_{h} q_{h}\frac{I_{h}}{N_{h}}+x_{b} q_{b}\frac{I_{b}}{N_{b}}+x_{dog} q_{dog}\frac{I_{dog}}{N_{dog}}{)S}_{v}- \varphi_{v}E_{v}-\mu_{v}E_{v} \\ \frac{dI_{v}}{dt}= \varphi_{v}E_{v}-\mu_{v}I_{v} \end{matrix} \right.$$

$$\left\{ \begin{matrix} \frac{dE_{h}}{dt}=b x_{h} p_{h}\frac{I_{v}}{N_{h}}S_{h}- \varphi_{h}E_{h}-\mu_{h}E_{h} \\ \frac{dI_{h}}{dt}= \varphi_{h}E_{h}-\gamma_{h}I_{h}-\mu_{h}I_{h} \end{matrix} \right.$$

$$\left\{ \begin{matrix} \frac{dE_{b}}{dt}=b x_{b} p_{b}\frac{I_{v}}{N_{b}}S_{b}- \varphi_{b}E_{b}-\mu_{b}E_{b} \\ \frac{dI_{b}}{dt}= \varphi_{b}E_{b}-\gamma_{b}I_{b}-\mu_{b}I_{b} \end{matrix} \right.$$

$$\left\{ \begin{matrix} \frac{dE_{dog}}{dt}=b x_{dog} p_{dog}\frac{I_{v}}{N_{dog}}S_{dog}- \varphi_{dog}E_{dog}-\mu_{dog}E_{dog} \\ \frac{dI_{dog}}{dt}= \varphi_{dog}E_{dog}-\gamma_{dog}I_{dog}-\mu_{dog}I_{dog} \end{matrix} \right.$$

With: *p*=pigs, *s*=sows, *d*=ducks, *c*=chickens, *v*=*Culex spp*, *h*=humans, *b*=bovines (cattle).

1. For each species, we identified transmission and migration terms, from which we deducted two vectors: $F$ is the rate at which new infections arise (transmission terms) and $V$ is the rate at which individuals enter or leave the compartment due to completion of an infection stage or death (migration terms).

$$F=\begin{matrix} b x_{p} p_{p}\frac{I_{v}}{N_{p}}S_{p} \\ 0 \\ b x_{s} p_{p}\frac{I_{v}}{N_{s}}S_{s} \\ 0 \\ b x_{d} p_{d}\frac{I_{v}}{N_{d}}S_{d} \\ 0 \\ b x_{c} p_{c}\frac{I_{v}}{N_{c}}S_{c} \\ 0 \\ b x_{p} q_{p}\frac{I_{p}}{N_{p}}S_{v}+b x_{s} q_{p}\frac{I_{s}}{N_{s}}S_{v}+b x_{d} q_{d}\frac{I_{d}}{N_{d}}S_{v}+b x_{c} q_{c}\frac{I_{c}}{N_{c}}S_{v}+b x_{h} q_{h}\frac{I_{h}}{N_{h}}S_{v}+b x_{b} q_{b}\frac{I_{b}}{N_{b}}S_{v}+b x_{dog} q_{dog}\frac{I_{dog}}{N_{dog}}S_{v} \\ 0 \\ b x_{h} p_{h}\frac{I_{v}}{N_{h}}S_{h} \\ 0 \\ b x_{b} p_{b}\frac{I_{v}}{N_{b}}S_{b} \\ 0 \\ b x_{dog} p_{dog}\frac{I_{v}}{N_{dog}}S_{dog} \\ 0 \end{matrix}$$

$$V=\begin{matrix} \varphi_{p}E_{p}+\mu_{p}E_{p} \\ {-\varphi}_{p}E_{p}+\gamma_{p}I_{p}+\mu_{p}I_{p} \\ \varphi_{p}E_{s}+\mu_{s}E_{s} \\ {-\varphi}_{p}E_{s}+\gamma_{p}I_{s}+\mu_{s}I_{s} \\ \varphi_{d}E_{d}+\mu_{d}E_{d} \\ -\varphi_{d}E_{d}+\gamma_{d}I_{d}+\mu_{d}I_{d} \\ \varphi_{c}E_{c}+\mu_{c}E_{c} \\ {-\varphi}_{c}E_{c}+\gamma_{c}I_{c}+\mu_{c}I_{c} \\ \varphi_{v}E_{v}+\mu_{v}E_{v} \\ -\varphi_{v}E_{v}+\mu_{v}I_{v} \\ \varphi_{h}E_{h}+\mu_{h}E_{h} \\ -\varphi_{h}E_{h}+\gamma_{h}I_{h}+\mu_{h}I_{h} \\ \varphi_{b}E_{b}+\mu_{b}E_{b} \\ {-\varphi}_{b}E_{b}+\gamma_{b}I_{b}+\mu_{b}I_{b} \\ \varphi_{dog}E_{dog}+\mu_{dog}E_{dog} \\ {-\varphi}_{dog}E_{dog}+\gamma_{dog}I_{dog}+\mu_{dog}I_{dog} \end{matrix}$$

2. We then defined the Jacobian Matrices at disease-free equilibrium DFE ($x_{0}$), where $S_{i}=N_{i}$ for both hosts and vectors: **F** $=\frac{\partial F_{i}\left( x_{0} \right)}{\partial x_{j}}$ and **V** $=\frac{\partial V_{i}\left( x_{0} \right)}{\partial x_{j}}$ , *i* and *j* being each species in the compartments corresponding to infected individuals (see page 4).
3. Thus, **F**.**V** ^-1^ is the NGM and R_0_ is the spectral radius of NGM, *i.e.* the maximal absolute value of all the eigenvalues of NGM.

**F** = $\begin{matrix} 0 & 0 & 0 & 0 & 0 & 0 & 0 & 0 & 0 & bx_{p}p_{p} & 0 & 0 & 0 & 0 & 0 & 0 \\ 0 & 0 & 0 & 0 & 0 & 0 & 0 & 0 & 0 & 0 & 0 & 0 & 0 & 0 & 0 & 0 \\ 0 & 0 & 0 & 0 & 0 & 0 & 0 & 0 & 0 & bx_{s}p_{s} & 0 & 0 & 0 & 0 & 0 & 0 \\ 0 & 0 & 0 & 0 & 0 & 0 & 0 & 0 & 0 & 0 & 0 & 0 & 0 & 0 & 0 & 0 \\ 0 & 0 & 0 & 0 & 0 & 0 & 0 & 0 & 0 & bx_{d}p_{d} & 0 & 0 & 0 & 0 & 0 & 0 \\ 0 & 0 & 0 & 0 & 0 & 0 & 0 & 0 & 0 & 0 & 0 & 0 & 0 & 0 & 0 & 0 \\ 0 & 0 & 0 & 0 & 0 & 0 & 0 & 0 & 0 & bx_{c}p_{c} & 0 & 0 & 0 & 0 & 0 & 0 \\ 0 & 0 & 0 & 0 & 0 & 0 & 0 & 0 & 0 & 0 & 0 & 0 & 0 & 0 & 0 & 0 \\ 0 & bx_{p}q_{p}N_{v}/N_{p} & 0 & bx_{s}q_{p}N_{v}/N_{s} & 0 & bx_{d}q_{d}N_{v}/N_{d} & 0 & bx_{c}q_{c}N_{v}/N_{c} & 0 & 0 & 0 & bx_{h}q_{h}N_{v}/N_{h} & 0 & bx_{b}q_{b}N_{v}/N_{b} & 0 & bx_{dog}q_{dog}N_{v}/N_{dog} \\ 0 & 0 & 0 & 0 & 0 & 0 & 0 & 0 & 0 & 0 & 0 & 0 & 0 & 0 & 0 & 0 \\ 0 & 0 & 0 & 0 & 0 & 0 & 0 & 0 & 0 & bx_{h}p_{h} & 0 & 0 & 0 & 0 & 0 & 0 \\ 0 & 0 & 0 & 0 & 0 & 0 & 0 & 0 & 0 & 0 & 0 & 0 & 0 & 0 & 0 & 0 \\ 0 & 0 & 0 & 0 & 0 & 0 & 0 & 0 & 0 & bx_{b}p_{b} & 0 & 0 & 0 & 0 & 0 & 0 \\ 0 & 0 & 0 & 0 & 0 & 0 & 0 & 0 & 0 & 0 & 0 & 0 & 0 & 0 & 0 & 0 \\ 0 & 0 & 0 & 0 & 0 & 0 & 0 & 0 & 0 & bx_{dog}p_{dog} & 0 & 0 & 0 & 0 & 0 & 0 \\ 0 & 0 & 0 & 0 & 0 & 0 & 0 & 0 & 0 & 0 & 0 & 0 & 0 & 0 & 0 & 0 \end{matrix}$ DFE

**V =** $\begin{matrix} \varphi_{p}+\mu_{p} & 0 & 0 & 0 & 0 & 0 & 0 & 0 & 0 & 0 & 0 & 0 & 0 & 0 & 0 & 0 \\ -\varphi_{p} & \gamma_{p}+\mu_{p} & 0 & 0 & 0 & 0 & 0 & 0 & 0 & 0 & 0 & 0 & 0 & 0 & 0 & 0 \\ 0 & 0 & \varphi_{p}+\mu_{s} & 0 & 0 & 0 & 0 & 0 & 0 & 0 & 0 & 0 & 0 & 0 & 0 & 0 \\ 0 & 0 & -\varphi_{p} & \gamma_{p}+\mu_{s} & 0 & 0 & 0 & 0 & 0 & 0 & 0 & 0 & 0 & 0 & 0 & 0 \\ 0 & 0 & 0 & 0 & \varphi_{d}+\mu_{d} & 0 & 0 & 0 & 0 & 0 & 0 & 0 & 0 & 0 & 0 & 0 \\ 0 & 0 & 0 & 0 & -\varphi_{d} & \gamma_{d}+\mu_{d} & 0 & 0 & 0 & 0 & 0 & 0 & 0 & 0 & 0 & 0 \\ 0 & 0 & 0 & 0 & 0 & 0 & \varphi_{c}+\mu_{c} & 0 & 0 & 0 & 0 & 0 & 0 & 0 & 0 & 0 \\ 0 & 0 & 0 & 0 & 0 & 0 & -\varphi_{c} & \gamma_{c}+\mu_{c} & 0 & 0 & 0 & 0 & 0 & 0 & 0 & 0 \\ 0 & 0 & 0 & 0 & 0 & 0 & 0 & 0 & \varphi_{v}+\mu_{v} & 0 & 0 & 0 & 0 & 0 & 0 & 0 \\ 0 & 0 & 0 & 0 & 0 & 0 & 0 & 0 & -\varphi_{v} & \mu_{v} & 0 & 0 & 0 & 0 & 0 & 0 \\ 0 & 0 & 0 & 0 & 0 & 0 & 0 & 0 & 0 & 0 & \varphi_{h}+\mu_{h} & 0 & 0 & 0 & 0 & 0 \\ 0 & 0 & 0 & 0 & 0 & 0 & 0 & 0 & 0 & 0 & -\varphi_{h} & \gamma_{h}+\mu_{h} & 0 & 0 & 0 & 0 \\ 0 & 0 & 0 & 0 & 0 & 0 & 0 & 0 & 0 & 0 & 0 & 0 & \varphi_{b}+\mu_{b} & 0 & 0 & 0 \\ 0 & 0 & 0 & 0 & 0 & 0 & 0 & 0 & 0 & 0 & 0 & 0 & -\varphi_{b} & \gamma_{b}+\mu_{b} & 0 & 0 \\ 0 & 0 & 0 & 0 & 0 & 0 & 0 & 0 & 0 & 0 & 0 & 0 & 0 & 0 & \varphi_{dog}+\mu_{dog} & 0 \\ 0 & 0 & 0 & 0 & 0 & 0 & 0 & 0 & 0 & 0 & 0 & 0 & 0 & 0 & -\varphi_{dog} & \gamma_{dog}+\mu_{dog} \end{matrix}$ DFE

**References**

1. Ladreyt H, Auerswald H, Tum S, Ken S, Heng L, In S, et al. Comparison of Japanese Encephalitis Force of Infection in Pigs, Poultry and Dogs in Cambodian Villages. Pathogens. sept 2020;9(9):719.

2. Boyer S, Durand B, Yean S, Brengues C, Maquart PO, Fontenille D, et al. Host-Feeding Preference and Diel Activity of Mosquito Vectors of the Japanese Encephalitis Virus in Rural Cambodia. Pathogens. mars 2021;10(3):376.

3. Karna AK, Bowen RA. Experimental Evaluation of the Role of Ecologically-Relevant Hosts and Vectors in Japanese Encephalitis Virus Genotype Displacement. Viruses. janv 2019;11(1):32.

4. Gajanana A, Rajendran R, Samuel PP, Thenmozhi V, Tsai TF, Kimura-Kuroda J, et al. Japanese encephalitis in south Arcot district, Tamil Nadu, India: a three-year longitudinal study of vector abundance and infection frequency. J Med Entomol. nov 1997;34(6):651‑9.

5. Chapman GE, Sherlock K, Hesson JC, Blagrove MSC, Lycett GJ, Archer D, et al. Laboratory transmission potential of British mosquitoes for equine arboviruses. Parasit Vectors. 12 août 2020;13(1):413.

6. Gould DJ, Barnett HC, Suyemoto W. Transmission of Japanese encephalitis virus by Culex gelidus theobald. Transactions of the Royal Society of Tropical Medicine and Hygiene. 1962;56(5).

7. van den Hurk AF, Nisbet DJ, Hall RA, Kay BH, MacKenzie JS, Ritchie SA. Vector competence of Australian mosquitoes (Diptera: Culicidae) for Japanese encephalitis virus. J Med Entomol. janv 2003;40(1):82‑90.

8. Cleton NB, Bosco-Lauth A, Page MJ, Bowen RA. Age-Related Susceptibility to Japanese Encephalitis Virus in Domestic Ducklings and Chicks. Am J Trop Med Hyg. 5 févr 2014;90(2):242‑6.

9. Di D, Li C, Zhang J, Hameed M, Wang X, Xia Q, et al. Experimental Infection of Newly Hatched Domestic Ducklings via Japanese Encephalitis Virus-Infected Mosquitoes. Pathogens. mai 2020;9(5):371.

10. Ricklin ME, Garcìa-Nicolàs O, Brechbühl D, Python S, Zumkehr B, Posthaus H, et al. Japanese encephalitis virus tropism in experimentally infected pigs. Vet Res. 2016;47(1).

11. Cappelle J, Duong V, Pring L, Kong L, Yakovleff M, Prasetyo DB, et al. Intensive Circulation of Japanese Encephalitis Virus in Peri-urban Sentinel Pigs near Phnom Penh, Cambodia. PLoS Negl Trop Dis. 7 déc 2016;10(12):e0005149.

12. Boyer S, Peng B, Pang S, Chevalier V, Duong V, Gorman C, et al. Dynamics and diversity of mosquito vectors of Japanese encephalitis virus in Kandal province, Cambodia. J Asia-Pac Entomol. 1 déc 2020;23(4):1048‑54.

13. van den Driessche P, Watmough J. Reproduction numbers and sub-threshold endemic equilibria for compartmental models of disease transmission. Math Biosci. 1 nov 2002;180(1):29‑48.
