# Supplementary material for: Modelling Japanese encephalitis virus transmission dynamics and human exposure in a Cambodian rural multi-host system
Source: PLoS Negl Trop Dis. 2022 Jul 11;16(7):e0010572. doi: 10.1371/journal.pntd.0010572 (PMC9302853; doi:10.1371/journal.pntd.0010572)
Supplement: S2 File — (DOCX) [file pntd.0010572.s002.docx]

**S2 File: Morris sensitivity analysis of R_0_ to parameter values (I), sensitivity analysis of model parameter estimates, R_0_ and human exposure indicators to host-to-vector transmission probability (*q_p_*, *q_d_*, *q_c_*), to *Culex spp* feeding preference for humans (*π_h_*), and to +/-20% seasonal variations of vector population size (*ψ*=0.2) in traditional villages of the three studied districts (II), and sensitivity analysis of R_0_ and human exposure indicators to *q_p_*, *q_d_*, *q_c_*, *π_h_*, and *ψ*, for the 3 variations of host community composition (III).**

1. **Morris sensitivity analysis of R_0_ to parameter values**

***Method***

To sort the model parameters according to their influence on R_0_, we first performed a global sensitivity analysis of R_0_ to model parameter values, using Morris method (Morris 1991). Model parameter effects can be (i) negligible, (ii) linear or (iii) non-linear or involved in interactions with other parameters. Morris method of global sensitivity analysis is a “one at a time” plan where only one parameter is modified at each simulation. The 51 parameter values of the model were discretized into 6 levels (+/- 25% of the initial value). The experimental design consisted of a random draw of 100 points on a regular grid of dimension 51 and 6 levels. It created 100 trajectories of 51+1 points by increasing or decreasing each component of the vector of parameters by one level in turn. For each parameter we computed:

- the sensitivity index, μ*, which measures the overall influence of the parameter on R_0_: it is the average of the absolute values of the variations of R_0_ associated with variations of the parameter values,
- the standard deviation of the elementary effects of the parameter, σ, which quantifies interactions between the parameter considered and the others, as well as the existence of non-linear effects of variations of the parameter values.

We used the package “sensitivity” (The R Core Team 2020) of R (version 4.0.2).

***Results***

The sensitivity analysis showed that among the 51 model parameters, 9 had a significant influence on R_0_, with μ* > 0.1 (Fig A). These were mostly vector- and pig-related parameters. Biting rate *b* was the most influent parameter, as it is a multiplicative factor in the force of infection of vectors and hosts. Similarly, the next most influential parameters *µ_v_* and *N_v_* strongly condition the force of infection of hosts as they are directly related to the quantity of infected mosquitoes *I_v_*. *γ_p_* was also influent as the longer pigs are viremic, the more mosquitoes feeding on them will become infected. *q_p_*, *p_p_*, and *π_p_* also condition the infection rate of the vectors. These 9 parameters displayed a low value of σ, showing that they had linear effects on R_0_ value. The values of σ and μ* were very small (<0.05) for the 42 remaining parameters, meaning that they had negligible effects on R_0_.


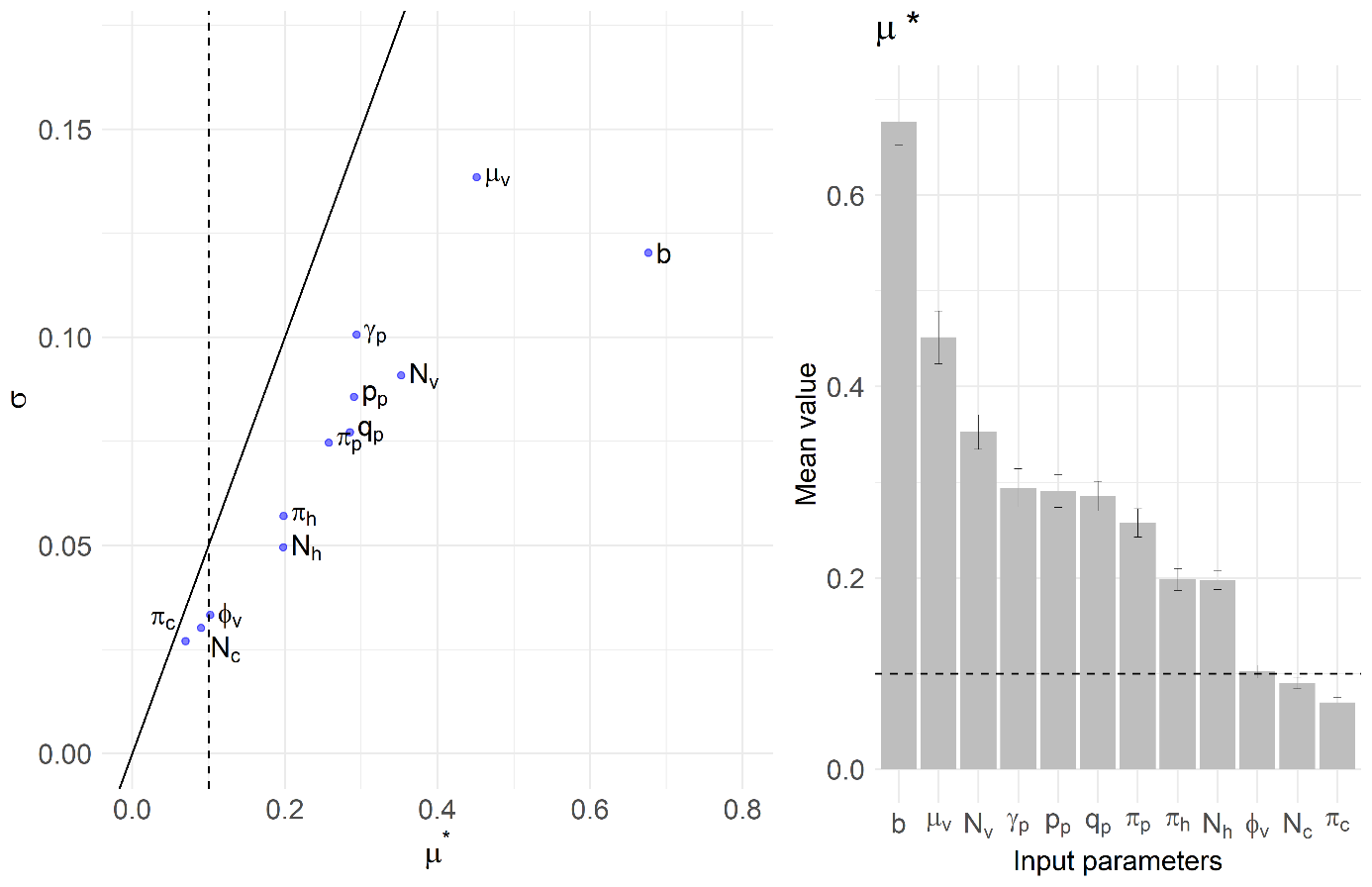


**Fig A. Morris plot (left) and µ* values (right) of the 9 more influential parameters on R_0_**. Other parameters had negligible effect on R_0_ (µ*<0.05, σ<0.05). The 0.5 slope line on Morris plot represents the limit between parameters whose effects are non-linear or include interaction with other factors, and parameters with a linear effect. The dashed line represents µ*=0.1.

1. **Sensitivity analysis of model parameter estimates, R_0_ and human exposure indicators to host-to-vector transmission probability (Table A), to *Culex spp.* feeding preference for humans (Table B), and to +/-20% seasonal variations of vector population size (Table C) in traditional villages of the three studied districts.**

Variations of the host-to-vector transmission probability (*q_p_*, *q_d_* and *q_c_*) had a very little impact on the estimated values of the feeding preferences of *Culex spp.* for ducks and dogs (*π_duck_* and *π_dog_*) (Table A). Estimated vector population sizes were impacted in D2 and D3 when *q_p_* was changed: a 90% decrease in the value of *q_p_* led to an *Nv*_2_ and *Nv*_3_ approximately three times larger than those obtained using the default setting, and a 90% increase in *q_p_* led to their slight decrease. The impact of changing the other parameters in these two districts, as well as changing the whole set of parameters in D1 on the estimated vector population sizes was not significant, with all 95% CIs of the new estimates overlapping with the estimates obtained with the default parameterization.

A decrease in the feeding preference of vectors for humans parameter, *π_h_*, had no impact on the estimated *π_duck_* and *π_dog_* but made *Nv*_1_, *Nv*_2_ and *Nv*_3_ slightly decreased with 95% CIs overlapping with those of the initial estimations (Table B).

R_0_ calculated in the traditional host community composition of each district observed in the field, given in Table 1 (main text), were not significantly affected by the new input parameter values and estimates (Table A & Table B), as well as annual exposure probability and average age at infection when computed with the new values of *q_p_*, *q_d_* and *q_c_* (Table A). However, and as expected, a decrease in *π_h_*, representing a lower exposure of people to vector bites, resulted in a lower annual probability of exposure and a higher average age at infection, especially in district D1 where the average age at infection reached 20.5 years when *π_h_* was halved (Table B).

**Table A: Impact of +/-90% variations in host-to-vector transmission probability (q_p_, q_d_, q_c_) on model parameter estimates, on R_0_, and on human exposure indicators (brackets: 95% confidence intervals), in traditional villages of the three studied districts.**

| **District** | **Parameter** | ***N_v_***^a^ | ***π_duck_***^b^ | ***π_dog_***^b^ | **R_0_** (95% CI) | **Annual exposure probability** (95% CI) | **Average age at infection** (95% CI) |
| --- | --- | --- | --- | --- | --- | --- | --- |
| **D1** | *q_p_* -90% | 25369 (13419-47964) | 0.43 (0.22-0.84) | 0.12 (0.08-0.20) | 1.07 (0.99-1.20) | 0.09 (<10^-5^-0.33) | 10.6 (2.9->100) |
|  | *q_p_* +90% | 13744 (10772- 17535) | 0.43 (0.22-0.83) | 0.12 (0.08-0.20) | 1.07 (1.03-1.22) | 0.09 (0.03-0.26) | 10.4 (3.8-29.3) |
|  | *q_d_* -90% | 30994 (25636- 37472) | 0.43 (0.22-0.83) | 0.12 (0.08-0.20) | 1.06 (0.99-1.14) | 0.10 (<10^-5^-0.21) | 10.4 (4.6->100) |
|  | *q_d_* +90% | 12618 (7531-20809) | 0.43 (0.23-0.81) | 0.12 (0.08-0.20) | 1.08 (1.00-1.20) | 0.09 (0.003-0.31) | 10.5 (3.1->100) |
|  | *q_c_* -90% | 19322 (12740-29304) | 0.43 (0.23-0.82) | 0.12 (0.08-0.20) | 1.08 (0.98-1.21) | 0.09 (<10^-5^-0.30) | 10.4 (3.0->100) |
|  | *q_c_* +90% | 16562 (12211-22464) | 0.43 (0.23-0.82) | 0.12 (0.08-0.20) | 1.07 (1.01-1.19) | 0.09 (0.01-0.31) | 10.4 (3.1->100) |
| **D2** | *q_p_* -90% | 172123 (145199-204041) | 0.43 (0.22-0.84) | 0.12 (0.08-0.20) | 1.21 (1.14-1.38) | 0.34 (0.23-0.48) | 2.8 (1.9-4.3) |
|  | *q_p_* +90% | 31015 (26022-36966) | 0.43 (0.22-0.83) | 0.12 (0.08-0.20) | 1.26 (1.17-1.36) | 0.34 (0.21-0.45) | 2.8 (2.1-4.7) |
|  | *q_d_* -90% | 54299 (45768-64419) | 0.43 (0.22-0.83) | 0.12 (0.08-0.20) | 1.25 (1.17-1.37) | 0.34 (0.23-0.48) | 2.8 (2.0-4.2) |
|  | *q_d_* +90% | 50756 (42725-60297) | 0.43 (0.23-0.81) | 0.12 (0.08-0.20) | 1.26 (1.17-1.39) | 0.34 (0.22-0.45) | 2.8 (2.1-4.4) |
|  | *q_c_* -90% | 56416 (47156-67494) | 0.43 (0.23-0.82) | 0.12 (0.08-0.20) | 1.27 (1.16-1.41) | 0.34 (0.20-0.48) | 2.8 (1.9-5.0) |
|  | *q_c_* +90% | 49038 (41821-57500) | 0.43 (0.23-0.82) | 0.12 (0.08-0.20) | 1.24 (1.15-1.34) | 0.34 (0.21-0.46) | 2.8 (2.0-4.6) |
| **D3** | *q_p_* -90% | 189014 (146783-243395) | 0.43 (0.22-0.84) | 0.12 (0.08-0.20) | 1.34 (1.27-1.49) | 0.48 (0.35-0.65) | 2.0 (1.4-2.7) |
|  | *q_p_* +90% | 35548 (30102-41980) | 0.43 (0.22-0.83) | 0.12 (0.08-0.20) | 1.40 (1.28-1.51) | 0.48 (0.32-0.59) | 2.0 (1.6-3.0) |
|  | *q_d_* -90% | 62829 (54320-72670) | 0.43 (0.22-0.83) | 0.12 (0.08-0.20) | 1.39 (1.30-1.51) | 0.48 (0.36-0.61) | 2.0 (1.5-2.7) |
|  | *q_d_* +90% | 56897 (46648-69397) | 0.43 (0.23-0.81) | 0.12 (0.08-0.20) | 1.40 (1.30-1.54) | 0.48 (0.34-0.62) | 2.0 (1.4-2.8) |
|  | *q_c_* -90% | 63859 (53052-76869) | 0.43 (0.23-0.82) | 0.12 (0.08-0.20) | 1.41 (1.31-1.55) | 0.48 (0.36-0.61) | 2.0 (1.5-2.7) |
|  | *q_c_* +90% | 56078 (47673-65964) | 0.43 (0.23-0.82) | 0.12 (0.08-0.20) | 1.37 (1.30-1.50) | 0.48 (0.37-0.63) | 2.0 (1.4-2.6) |

^a^Vector population size (number of mosquitoes). ^b^Feeding preferences relative to pigs, used as a reference

**Table B: Impact of a decrease in Culex spp. feeding preference for humans (π_h_) on model parameter estimates, on R_0_, and on human exposure indicators (brackets: 95% confidence intervals), in traditional villages of the three studied districts.**

| **District** | **Parameter** | ***N_v_***^a^ | ***π_duck_***^b^ | ***π_dog_***^b^ | **R_0_** | **Annual exposure probability** | **Average age at infection** |
| --- | --- | --- | --- | --- | --- | --- | --- |
| **D1** | *π_h_* -10% | 16825 (11815-23961) | 0.43 (0.22-0.84) | 0.12 (0.08-0.20) | 1.07 (0.99-1.20) | 0.08 (<10^-5^-0.28) | 11.7 (3.4->100 |
|  | *π_h_* -20% | 15887 (11216-22503) | 0.43 (0.22-0.85) | 0.12 (0.08-0.21) | 1.07 (0.99-1.20) | 0.08 (<10^-5^-0.24) | 13.1 (4.0->100) |
|  | *π_h_* -30% | 14989 (10753-20894) | 0.43 (0.22-0.83) | 0.12 (0.08-0.20) | 1.07 (0.99-1.19) | 0.07 (<10^-5^-0.22) | 14.9 (4.5->100) |
|  | *π_h_* -40% | 14111 (10189-19543) | 0.43 (0.22-0.85) | 0.12 (0.08-0.21) | 1.07 (1.0-1.20) | 0.06 (0.0003-0.19) | 17.3 (5.2->100) |
|  | *π_h_* -50% | 13259 (9732-18066) | 0.43 (0.22-0.83) | 0.12 (0.08-0.20) | 1.07 (1.01-1.21) | 0.05 (0.005-0.16) | 20.5 (6.0->100) |
| **D2** | *π_h_* -10% | 49530 (41890-58564) | 0.43 (0.22-0.84) | 0.12 (0.08-0.20) | 1.25 (1.16-1.37) | 0.31 (0.19-0.43) | 3.1 (2.2-5.1) |
|  | *π_h_* -20% | 46791 (39568-55332) | 0.43 (0.22-0.85) | 0.12 (0.08-0.21) | 1.25 (1.15-1.37) | 0.28 (0.15-0.40) | 3.5 (2.4-6.0) |
|  | *π_h_* -30% | 44122 (37311-52176) | 0.43 (0.22-0.83) | 0.12 (0.08-0.20) | 1.25 (1.16-1.36) | 0.25 (0.15-0.36) | 3.9 (2.7-6.4) |
|  | *π_h_* -40% | 41535 (35119-49122) | 0.43 (0.22-0.85) | 0.12 (0.08-0.21) | 1.25 (1.16-1.37) | 0.22 (0.13-0.32) | 4.5 (3.0-7.6) |
|  | *π_h_* -50% | 39025 (32995-46157) | 0.43 (0.22-0.83) | 0.12 (0.08-0.20) | 1.25 (1.16-1.37) | 0.18 (0.11-0.27) | 5.3 (3.6-9.3) |
| **D3** | *π_h_* -10% | 56160 (47426-66501) | 0.43 (0.22-0.84) | 0.12 (0.08-0.20) | 1.39 (1.29-1.53) | 0.44 (0.31-0.56) | 2.1 (1.6-3.1) |
|  | *π_h_* -20% | 52874 (44490-62837) | 0.43 (0.22-0.85) | 0.12 (0.08-0.21) | 1.39 (1.28-1.52) | 0.40 (0.27-0.53) | 2.4 (1.7-3.6) |
|  | *π_h_* -30% | 49716 (42076-58745) | 0.43 (0.22-0.83) | 0.12 (0.08-0.20) | 1.39 (1.29-1.52) | 0.36 (0.25-0.48) | 2.7 (1.9-3.9) |
|  | *π_h_* -40% | 46643 (39405-55210) | 0.43 (0.22-0.85) | 0.12 (0.08-0.21) | 1.39 (1.28-1.52) | 0.32 (0.21-0.43) | 3.0 (2.2-4.8) |
|  | *π_h_* -50% | 43668 (37118-51373) | 0.43 (0.22-0.83) | 0.12 (0.08-0.20) | 1.39 (1.28-1.51) | 0.27 (0.18-0.37) | 3.6 (2.5-5.5) |

^a^Vector population size (number of mosquitoes). ^b^Feeding preferences relative to pigs, used as a reference

Incorporating seasonal variations of vector population size (*ψ*=0.2) resulted in a slight increase in estimated *Nv*_1_, *Nv*_2_ and *Nv*_3_, as well as a slight decrease in *π_duck_* and *π_dog_* values (Table C), with 95% CIs overlapping with those of the initial estimations. In district D1, the maximum value of R_0_ during the rainy season (*i.e.* when the vector population size is maximal) was greater than 1, and the lower bound of its CI was above 1 (Table C). The minimum value of R_0_ during the dry season (*i.e.* when the vector population size is minimal) was slightly below 1, but the upper bound of its 95% CI remained above 1. Furthermore, simulations showed that the dry season did not stop the circulation of JEV in D1, as the annual probability of human exposure during year 30 was 0.13 (the lower bound of the confidence interval was zero, however) (Table C). This suggests that in this district and with this range of seasonality in vector population size, JEV could keep circulating during the unfavorable season, *i.e.* the dry season, and that the multi-host system studied could constitute a maintenance community for JEV. In addition, the introduction of seasonality resulted in an increase in the annual probability of human exposure (between 17% and 44%, depending on the district), associated with a slight decrease in the average age at infection. It should be noted that incorporating seasonal variations of vector population size in the model did not enhance the model fit to the observed data, as the minimal log-likelihood was larger (227.1) than without seasonal variations (224.7).

**Table C: Impact of +/-20% seasonal variations of vector population size (****ψ=0.2) on model parameter estimates, R_0_, and human exposure indicators (brackets: 95% confidence intervals), in traditional villages of the three studied districts.**

| **District** | ***N_v_***^a^ | ***π_duck_***^b^ | ***π_dog_***^b^ | **R_0,_ dry season**^c^ | **R_0,_ rainy season**^c^ | **Annual exposure probability** | **Average age at infection** |
| --- | --- | --- | --- | --- | --- | --- | --- |
| **D1** | 19,042  (13,076-27,730) | 0.40  (0.21-0.80) | 0.10  (0.06-0.16) | 0.98  (0.89-1.12) | 1.20  (1.08-1.37) | 0.13  (<10^-5^-0.37) | 7.8  (2.4->100) |
| **D2** | 59,015  (47,330-73,585) | 0.40  (0.21-0.80) | 0.10  (0.06-0.16) | 1.19  (1.07-1.33) | 1.46  (1.31-1.63) | 0.44  (0.26-0.60) | 2.1  (1.5-3.8) |
| **D3** | 65,403  (54,368-78,678) | 0.40  (0.21-0.80) | 0.10  (0.06-0.16) | 1.31  (1.20-1.44) | 1.60  (1.47-1.76) | 0.55  (0.40-0.70) | 1.7  (1.2-2.4) |

^a^Vector population size (number of mosquitoes). ^b^Feeding preferences relative to pigs, used as a reference. ^c^Values of R_0_ when the vector population size is minimal (dry season), and maximal (rainy season).

1. **Sensitivity analysis of R_0_ and human exposure indicators to *q_p_*, *q_d_*, *q_c_*, *π_h_*, and *ψ*, for the 3 variations of host community composition.**

**Fig B:** **Impact of variations of input parameters (q_p_, q_d_, q_c_, π_h_, ψ) on output variables, in variation 1: relative share of competent hosts versus non-competent hosts BSA.** Thin dashed lines correspond to decreasing parameters (or R_0_ min in the case of **ψ** modification), dotted lines connecting crosses correspond to their increase (or R_0_ max in the case of **ψ** modification), and dotted/dashed lines (**ψ** modification plot) correspond to the consideration of a seasonality in vector abundance.


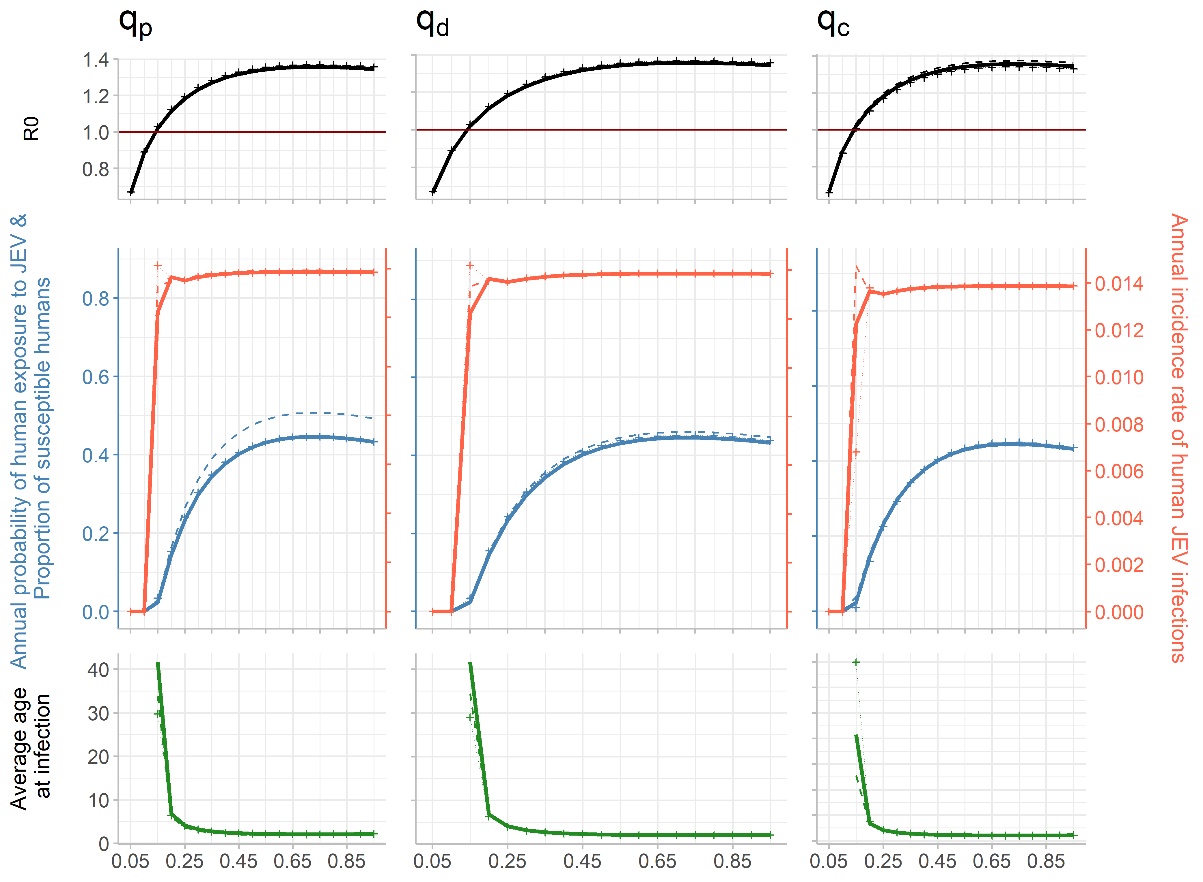

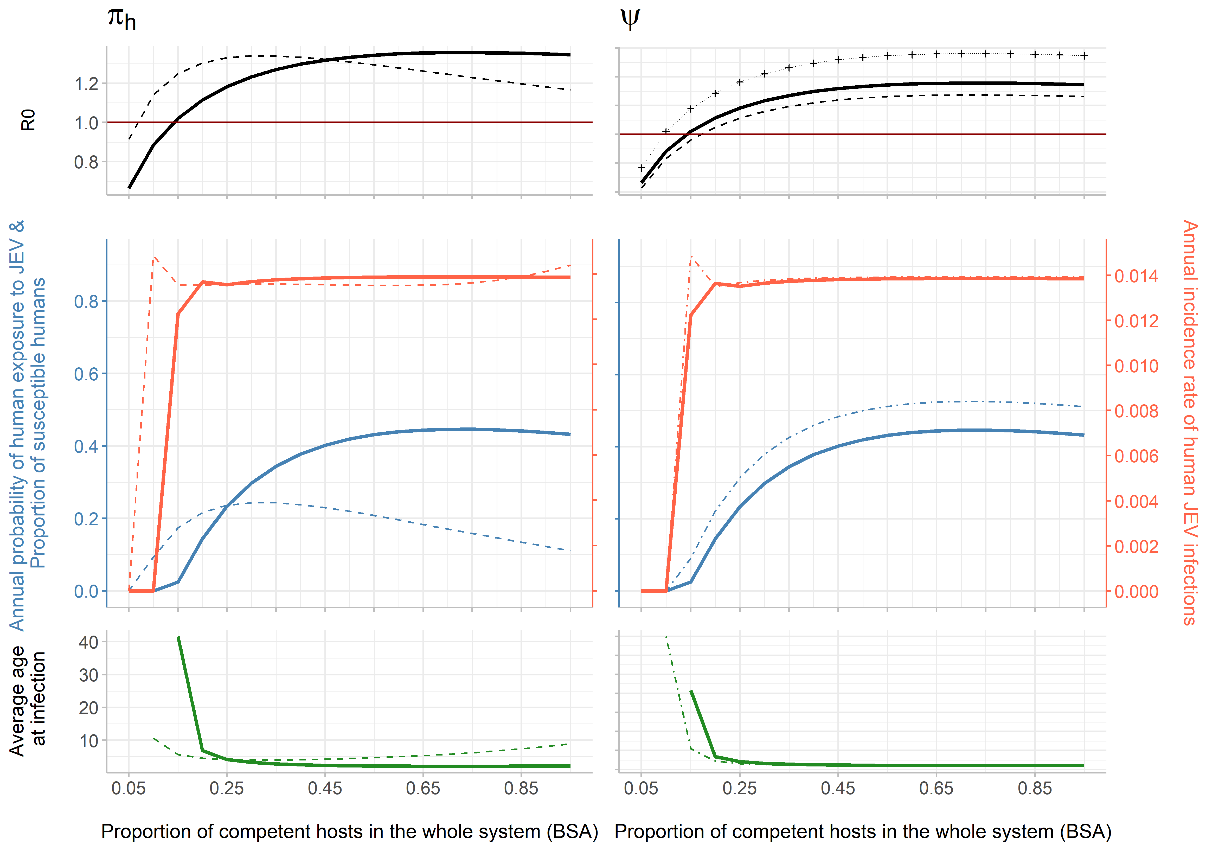


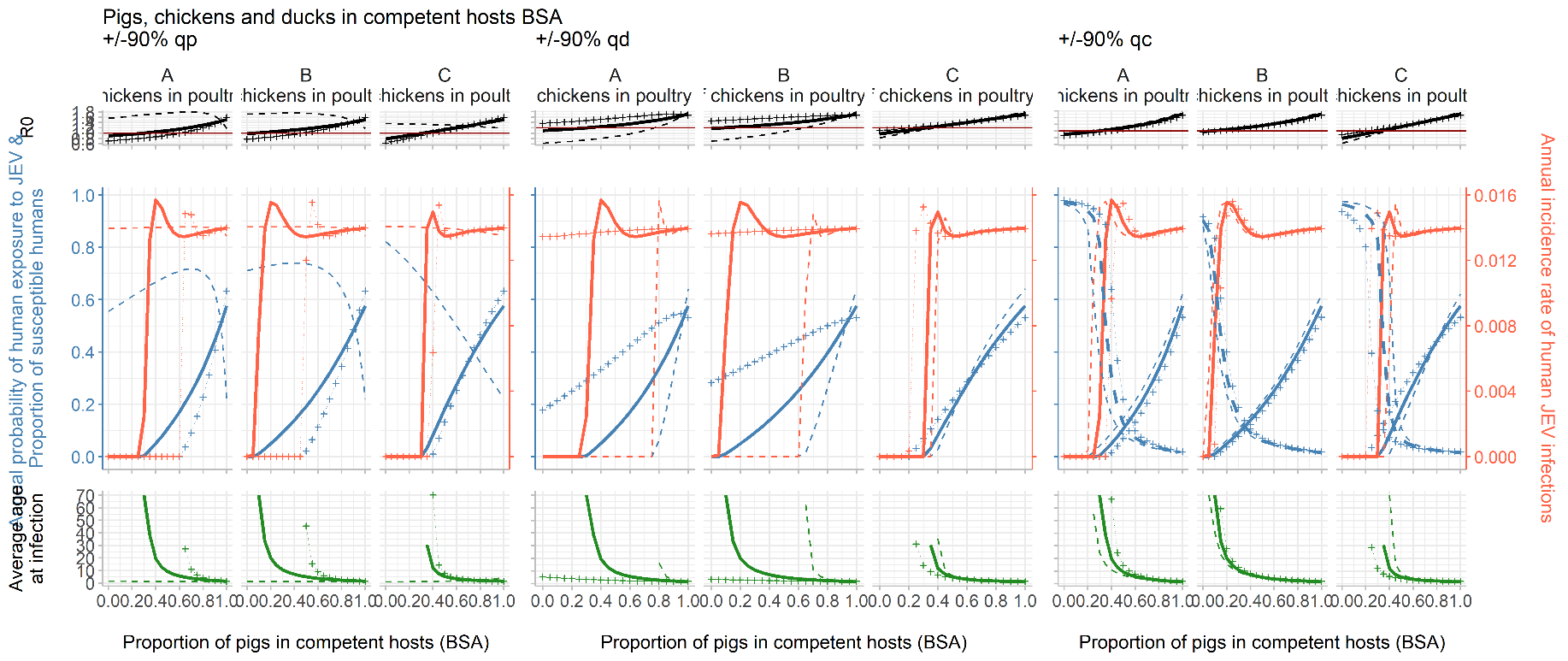


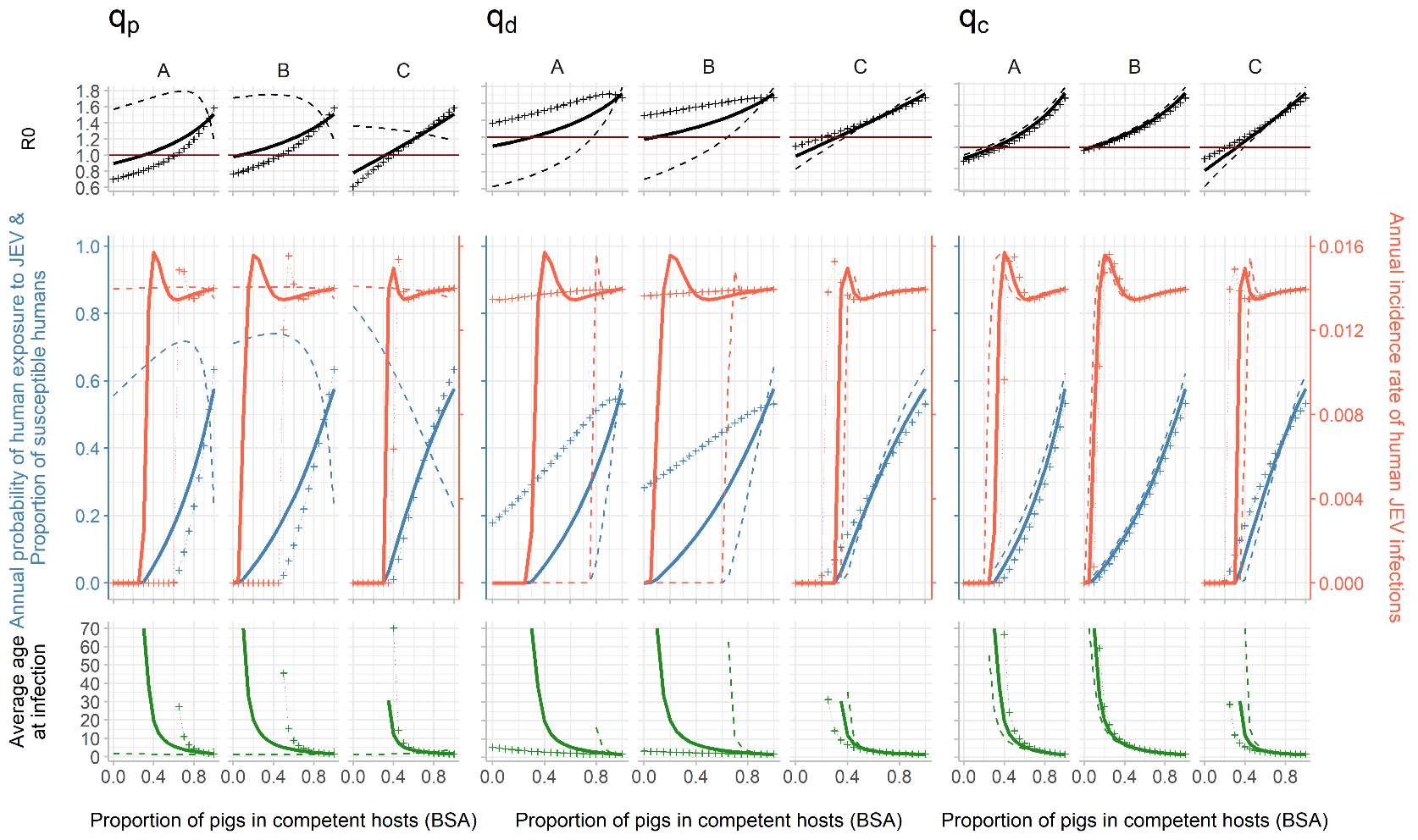

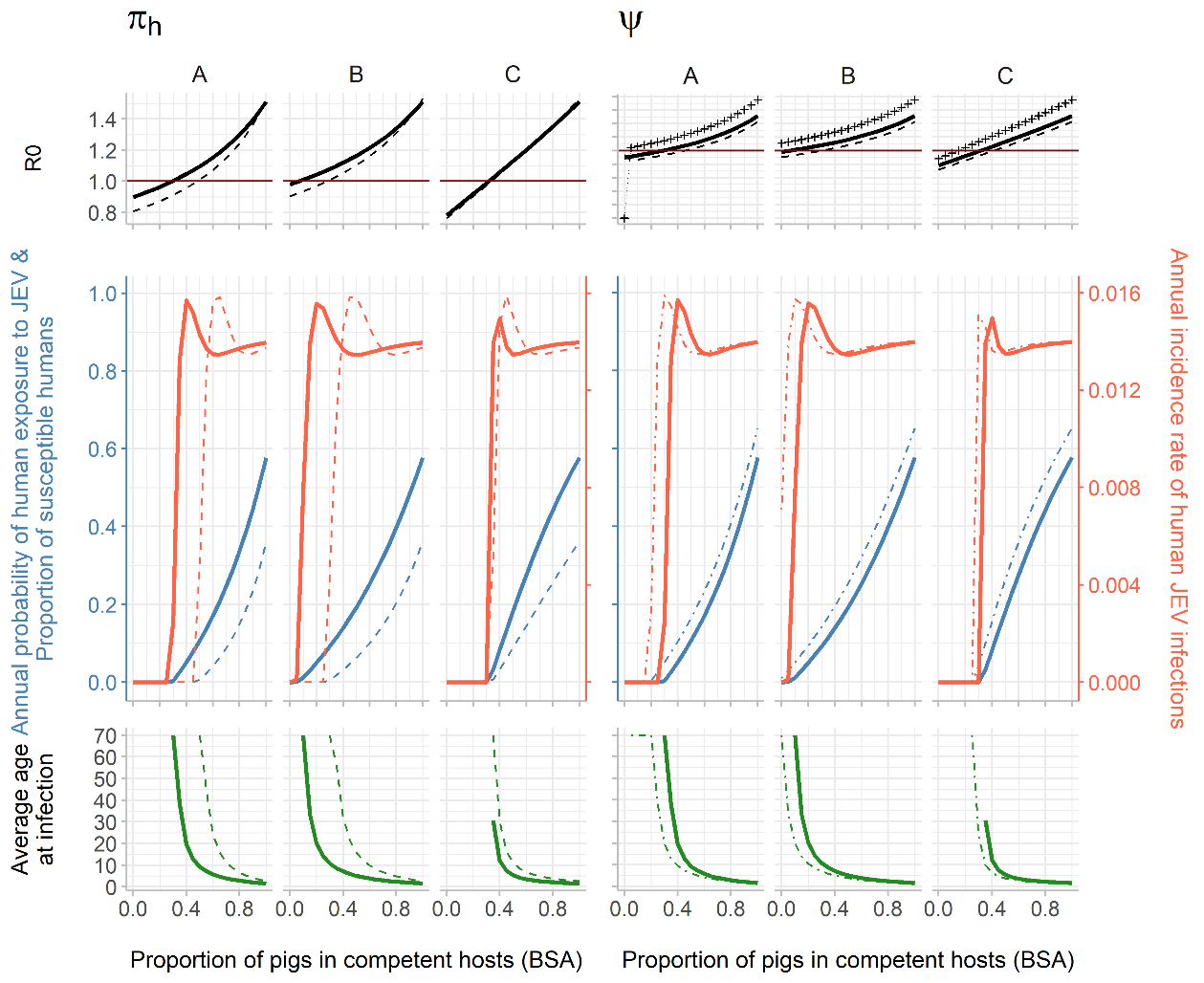


**Fig C:** **Impact of variations of input parameters (q_p_, q_d_, q_c_, π_h_, ψ) on output variables in variation 2: relative share of pigs, chickens and ducks among competent hosts BSA.** Thin dashed lines correspond to decreasing parameters (or R_0_ min in the case of **ψ** modification), dotted lines connecting crosses correspond to their increase (or R_0_ max in the case of **ψ** modification), and dotted/dashed lines (**ψ** modification plot) correspond to the consideration of a seasonality in vector abundance.


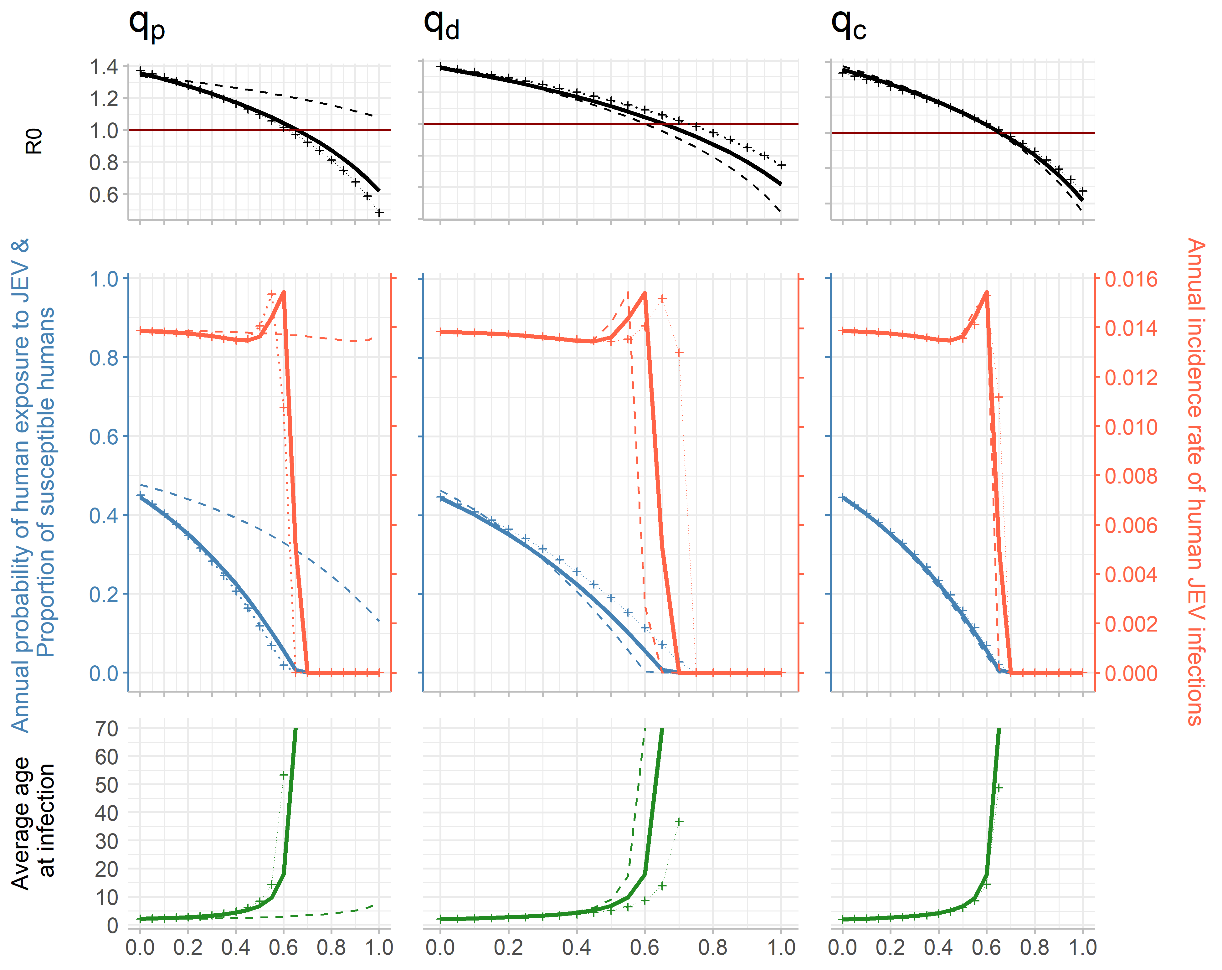

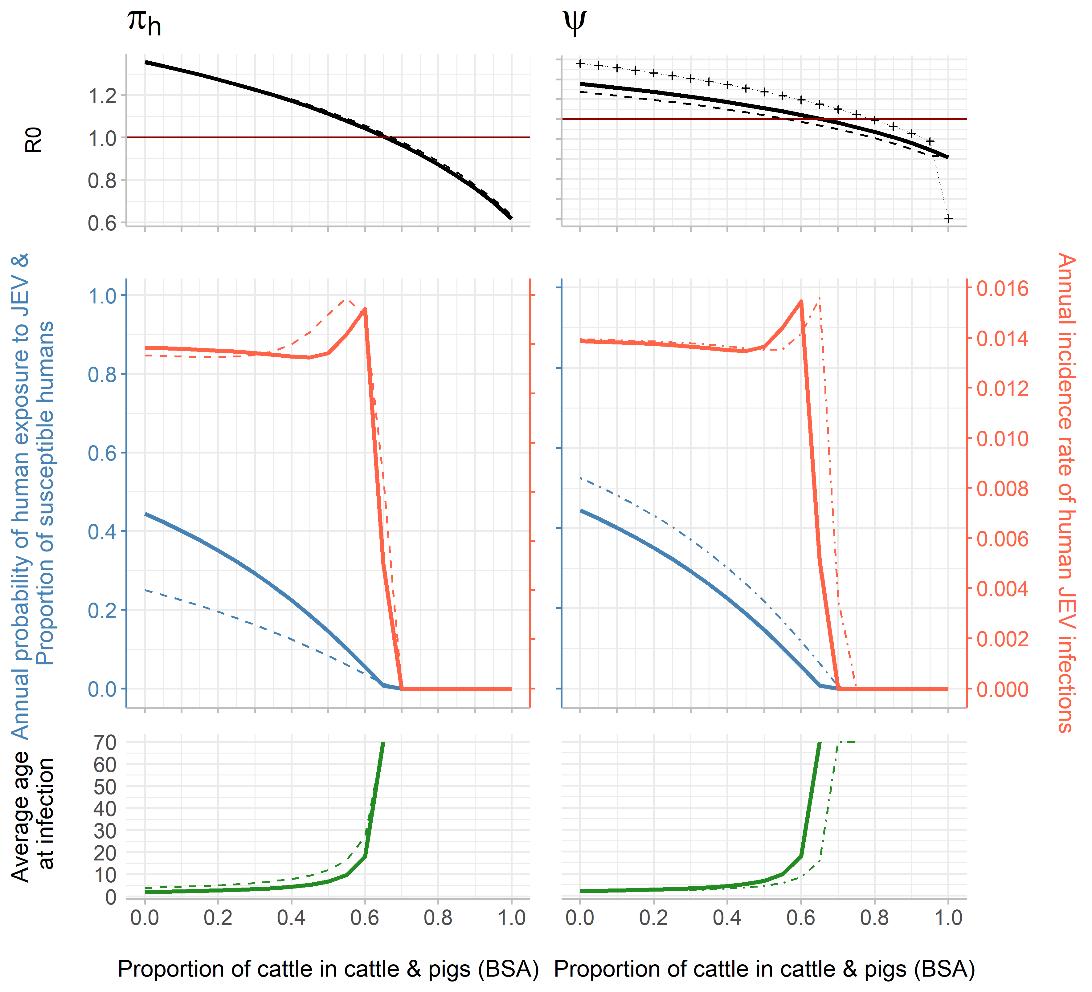


**Fig D:** **Impact of variations of input parameters (q_p_, q_d_, q_c_, π_h_, ψ) on output variables in variation 3: relative share of cattle among cattle and pigs BSA.** Thin dashed lines correspond to decreasing parameters (or R_0_ min in the case of **ψ** modification), dotted lines connecting crosses correspond to their increase (or R_0_ max in the case of **ψ** modification), and dotted/dashed lines (**ψ** modification plot) correspond to the consideration of a seasonality in vector abundance.
