# Supplementary figures and images for: Modelling Japanese encephalitis virus transmission dynamics and human exposure in a Cambodian rural multi-host system
Source: PLoS Negl Trop Dis. 2022 Jul 11;16(7):e0010572. doi: 10.1371/journal.pntd.0010572 (PMC9302853; doi:10.1371/journal.pntd.0010572)

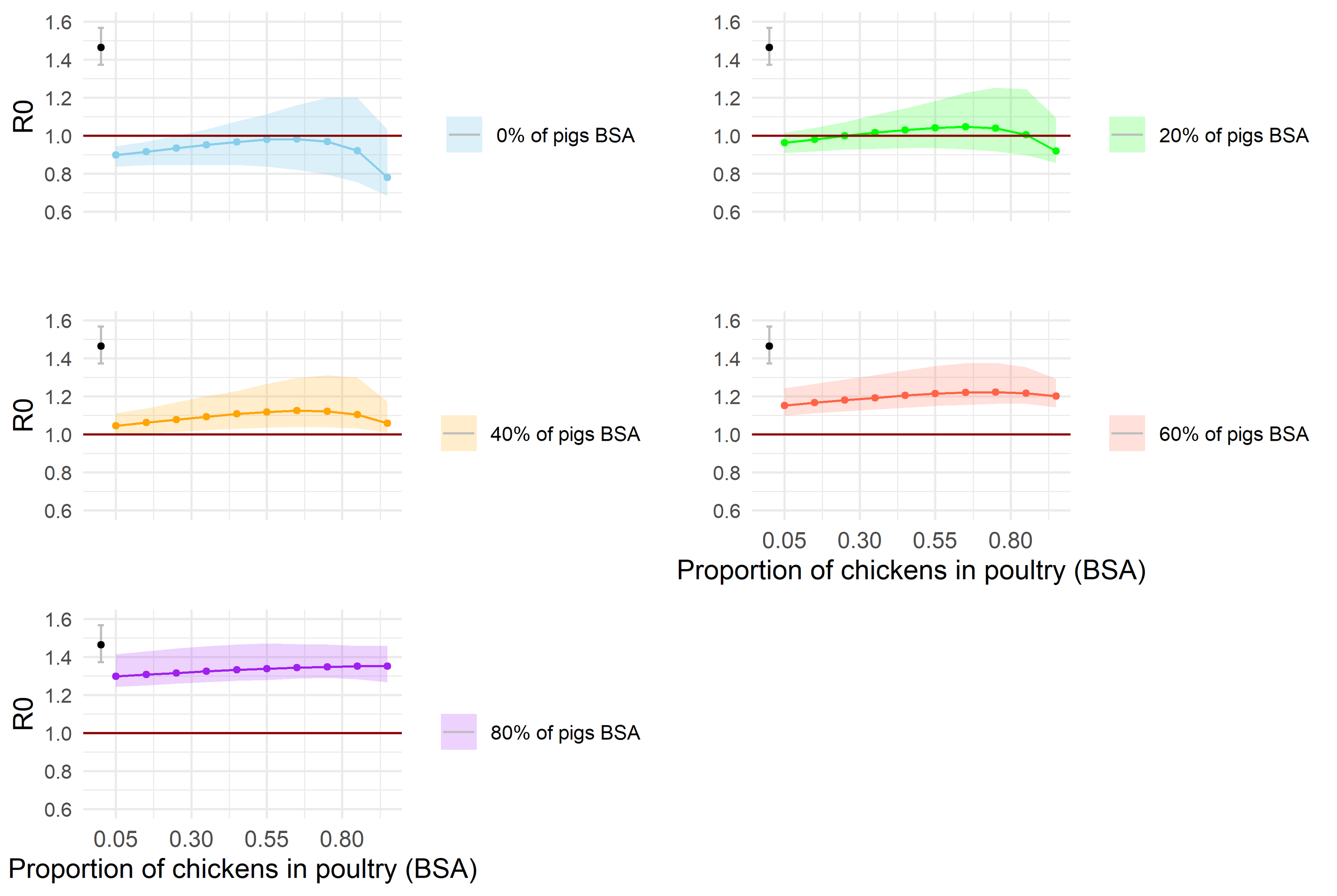

Supplement: S1 Fig — The black point corresponds to the R0 for 100% of pigs in competent hosts BSA. (TIF) [file pntd.0010572.s003.tif]

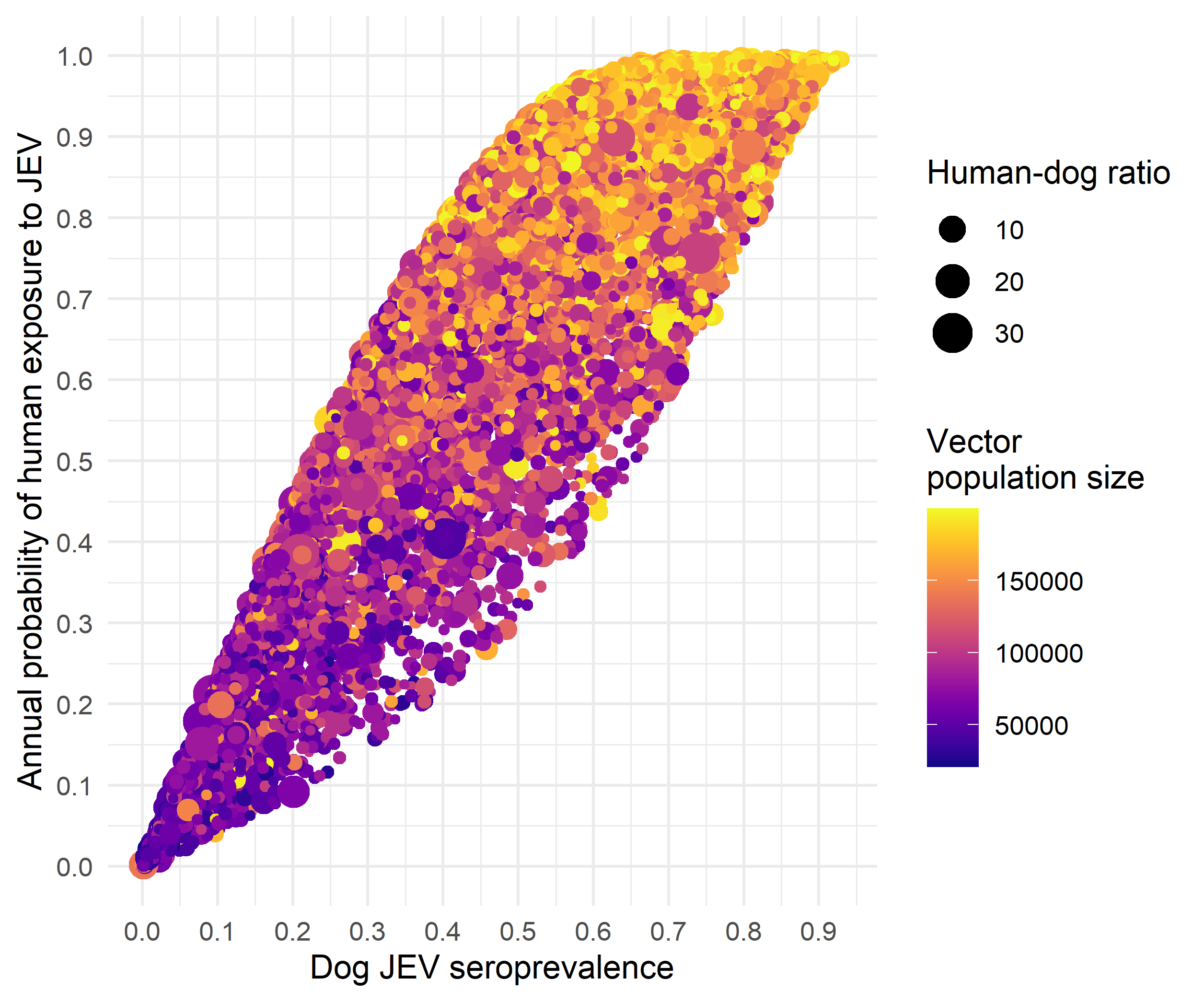

Supplement: S2 Fig — (TIF) [file pntd.0010572.s004.tif]
